# Improving implementation of school-based healthy eating and physical activity policies, practices, and programs: a systematic review

**DOI:** 10.1093/tbm/ibab037

**Published:** 2021-06-03

**Authors:** Courtney Barnes, Sam McCrabb, Fiona Stacey, Nicole Nathan, Sze Lin Yoong, Alice Grady, Rachel Sutherland, Rebecca Hodder, Christine Innes-Hughes, Marc Davies, Luke Wolfenden

**Affiliations:** 1 Hunter New England Population Health, Wallsend, New South Wales, Australia; 2 School of Medicine and Public Health, University of Newcastle, Callaghan, New South Wales, Australia; 3 Priority Research Centre for Health Behaviour, University of Newcastle, Callaghan, New South Wales, Australia; 4 Hunter Medical Research Institute, New Lambton, New South Wales, Australia; 5 Department of Nursing and Allied Health, Swinburne University of Technology, Melbourne, Victoria, Australia; 6 New South Wales Office of Preventive Health, Sydney, New South Wales, Australia

**Keywords:** School, Implementation, Nutrition, Physical activity, Policy

## Abstract

Although best practice recommendations exist regarding school-based healthy eating and physical activity policies, practices, and programs, research indicates that implementation is poor. As the field of implementation science is rapidly evolving, an update of the recent review of strategies to improve the implementation of healthy eating and physical activity interventions in schools published in the Cochrane Library in 2017 was required. The primary aim of this review was to examine the effectiveness of strategies that aim to improve the implementation of school‐based policies, practices, or programs to address child diet, physical activity, or obesity. A systematic review of articles published between August 31, 2016 and April 10, 2019 utilizing Cochrane methodology was conducted. In addition to the 22 studies included in the original review, eight further studies were identified as eligible. The 30 studies sought to improve the implementation of healthy eating (*n* = 16), physical activity (*n* = 11), or both healthy eating and physical activity (*n* = 3). The narrative synthesis indicated that effect sizes of strategies to improve implementation were highly variable across studies. For example, among 10 studies reporting the proportion of schools implementing a targeted policy, practice, or program versus a minimal or usual practice control, the median unadjusted effect size was 16.2%, ranging from –0.2% to 66.6%. Findings provide some evidence to support the effectiveness of strategies in enhancing the nutritional quality of foods served at schools, the implementation of canteen policies, and the time scheduled for physical education.

**Implications**

**Practice:** Findings of the review provide guidance for health promotion practitioners and jurisdictions working within school-based settings to implement World Health Organization obesity-prevention recommendations

**Policy:** The review identified that the implementation of several policies have facilitated improvements in the availability of healthy foods in schools, of magnitudes that could lead to substantial improvements in public health nutrition.

**Research**: The review builds on current literature to provide greater clarity on the effect of strategies to support the implementation of evidence-based healthy eating, physical activity, and obesity-prevention policies and practices, a critical component to achieve the public health benefits of such policies and practices.

## INTRODUCTION

An unhealthy diet, inadequate physical activity, and excessive weight gain are independent risk factors for the leading causes of death and disability globally, including cancer and cardiovascular disease [1]. In childhood and adolescents, a healthy diet [2, 3], physical activity [4–6], and healthy weight [7] have also been found to be independently associated with immediate positive health outcomes, including improved mental health and academic performance. Additionally, as health behaviors developed during childhood have been found to track into adulthood [8], interventions to address these risk factors are recommended by the World Health Organization (WHO) and governments internationally as part of population health and chronic disease prevention strategies [9].

Schools represent an attractive and important setting for health promotion initiatives as they provide continual access to children during a critical period of their development [10, 11]. Systematic reviews have identified well over 100 randomized trials of school-based interventions targeting student diet, physical activity, or obesity and have demonstrated that such interventions can be effective in reducing associated health risks [12–14]. On the basis of such evidence, national and international best-practice guidelines have been established acknowledging the potential for school-based settings to influence the development of children’s healthy eating and physical activity behaviors [15–18]. These evidence-based guidelines recommend schools adopt a range of policies, practices, and programs, such as the scheduling and provision of physical activity and active play opportunities, reducing the availability of unhealthy foods for sale at schools and alignment of foods services with national dietary guidelines [15, 18, 19].

Despite the existence of these best-practice guidelines, research suggests that schools fail to routinely implement evidence-based policies, practices, and programs. For example, the 2014 report card of physical activity in Ireland found that only 17% of primary schools were providing the mandated 2 hr of compulsory physical education per week [20]. Similarly, studies of Australian primary schools have found that only 5%–35% of Australian schools comply with mandated school canteen policies regarding the availability of unhealthy foods and beverages [21], whilst just 10% of middle and high schools within the USA prohibit the sale of sugar-sweetened beverages other than soda [22]. Without routine implementation, the public health benefits of such policies and practices promoting healthy eating and physical activity will not be fully achieved.

The field of implementation science seeks to address this issue through the generation of evidence to facilitate the use of evidence-based policies, practices, and programs [23]. Implementation science research seeks to identify effective strategies, such as educational outreach visits, reminders, or audit and feedback, which best support the integration of evidence-based practices into a specific setting [23, 24]. Implementation trials seek to assess the impact of such implementation strategies on the measures of the implementation of an evidence-based policy, practice, or program [23, 24]. Relative to trials testing the efficacy of behavioral interventions, few implementation trials have been conducted examining strategies to best implement evidence-based healthy eating, physical activity, or obesity-prevention interventions in the school setting [25].

We conducted a comprehensive Cochrane review on the topic in 2017, which included studies (randomized and nonrandomized) published until August 2016 [25]. The review identified 27 studies, 15 of which aimed to improve the implementation of healthy eating practices, and 6 studies targeted physical activity (the remaining studies pertained to alcohol and tobacco prevention) [25]. Findings of the review was mixed, with inconsistent improvements in the implementation of policies, practices, or programs reported across studies. Additionally, considerable clinical heterogeneity in the type of implementation strategies tested, policies, practices, and programs targeted, and outcomes assessed across the included studies was evident within the review [25]. Overall, the impact of strategies on the implementation of physical activity and healthy eating policies, practices, and programs was unclear, and the certainty of the evidence was low [25].

We are not aware of any review of school-based implementation interventions undertaken since that review. The field of implementation science, however, is rapidly evolving and a number of implementation studies targeting healthy eating and physical activity policies, practices, and programs have been published in recent years [26–28]. The addition of new studies may provide greater clarity regarding the effect of such strategies on the implementation of evidence-based policies, programs, and practices in schools given variable and inconclusive findings from the previous review. The aim of this review, therefore, was to update our previous review by Wolfenden et al. to reflect the current state of the evidence.

## OBJECTIVES

The primary aim of this review was to examine the effectiveness of strategies that aim to improve the implementation of school‐based policies, practices, or programs to address child diet, physical activity, or obesity.

## METHODS

This review aligns with the reporting guidelines specified within the 2009 PRISMA checklist for systematic reviews [29] (Supplementary File I) and utilized Cochrane methodology to replicate the previous review by Wolfenden et al. [25].

### Selection criteria

#### Types of studies

“Implementation” was defined as the use of strategies to adopt and integrate evidence‐based health interventions and to change practice patterns within specific settings [30]. Any study (randomized or nonrandomized) conducted at any scale with a parallel control group that compared a strategy to implement school-based policies, practices, or programs to address child diet, physical activity, overweight, or obesity by school staff to “no intervention,” “usual” practice, or a different implementation strategy was eligible for inclusion. Unlike the original review, we excluded studies solely targeting the implementation of tobacco or alcohol use prevention policies, practices, or programs as these were not the focus of this review update.

#### Types of interventions

Studies employing any strategy with the primary aim of improving implementation of healthy eating, physical activity, or obesity prevention policies, practices, or programs in schools were eligible. Strategies must have aimed to improve the implementation of policies, practices, or programs by usual school staff. Strategies could include quality improvement initiatives, education and training, performance feedback, prompts and reminders, implementation resources (e.g., manuals), financial incentives, penalties, communication and social marketing strategies, professional networking, the use of opinion leaders, implementation consensus processes, or other strategies [25].

#### Types of participants

Eligible studies were set in schools (e.g., primary, elementary, middle, and secondary) where the age of students is predominately between 5 and 18 years [31]. Study participants could be any stakeholders who may influence the uptake, implementation, or sustainability of the target health promoting policy, practice, or program in schools, including teachers, managers, cooks/catering staff, or other staff of schools and education departments.

#### Types of outcome measures

Studies with any objectively or subjectively (self‐reported) assessed measure of school policy, practice, or program implementation—including uptake, partial/complete uptake (e.g., consistent with protocol/design), or routine use—were included. Implementation could have occurred at any scale (e.g., local, national, or international). Implementation outcomes (e.g., frequency of practice implementation by teachers) must have been undertaken by a school or routine school personnel and not those undertaken by paid research personnel. Child-level outcomes (e.g., child diet and physical activity) were not considered as implementation outcomes. Studies collecting outcome data at follow-up only for an implementation outcome were included for randomized trial designs only (i.e., equivalent baseline values assumed or differ only by chance) or if baseline values were assumed to be zero (i.e., a school policy did not exist at baseline). Implementation outcome data may have been obtained from audits of school records, questionnaires or surveys of staff, direct observation or recordings, examination of routinely collected information from government departments (such as compliance with food standards or breaches of department regulations), or other sources.

### Search strategy

The original search by Wolfenden et al. was undertaken for studies published up to August 31, 2016 [25]. Small amendments were made to the original search strategy to improve the sensitivity of the search, which was conducted by an experienced research librarian. This updated review included eligible studies published up until April 10, 2019, from a search of the following electronic databases: Cochrane Library, including the Cochrane Central Register of Controlled Trials (CENTRAL); MEDLINE; MEDLINE InProcess & Other Non‐Indexed Citations; Embase Classic and Embase; PsycINFO; Education Resource Information Center; Cumulative Index to Nursing and Allied Health Literature; Dissertations and Theses; and SCOPUS (Appendix II Medline search strategy). Additionally, a search of the World Health Organization International Clinical Trials Registry Platform (www.who.int/ictrp/) and ClinicalTrials.gov (www.clinicaltrials.gov) conducted by Wolfenden et al. was replicated for this review. The “Characteristics of Ongoing Studies” section of the original review was also searched for potentially eligible studies that were unpublished at the time of the first review [25].

### Data collection and analysis

#### Selection of studies

**Fig 1:**
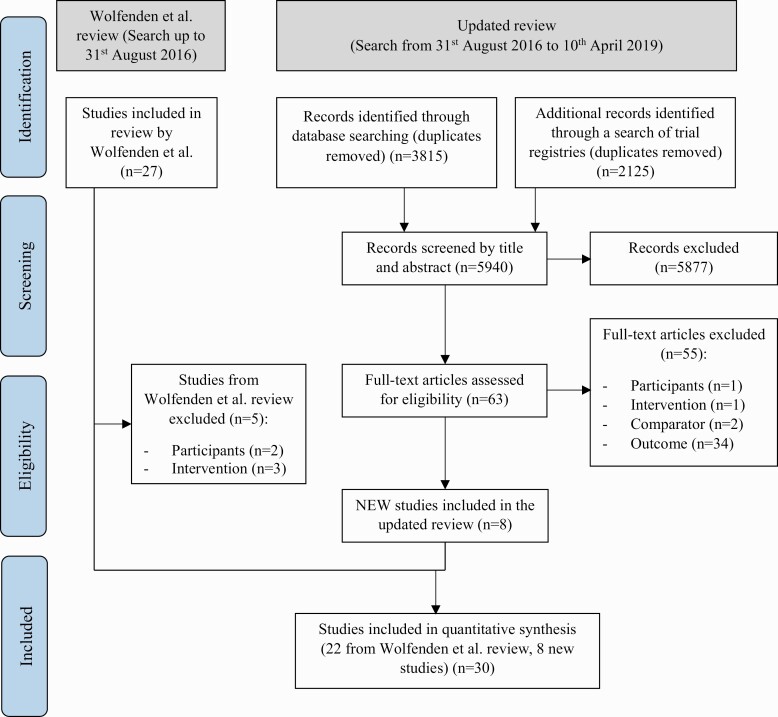
Study flow diagram.

Title and abstract screening for eligible studies was performed independently by review authors in pairs. Authors were not blind to author or journal information. For potentially eligible studies, full texts of manuscripts were examined for eligibility by a pair of review authors independently. Reasons for exclusion were documented for all studies and recorded in Fig. 1. Disagreements between review authors were resolved via consensus or, when required, by a third author.

#### Data extraction and management

Data extraction was completed independently by two authors unblinded to author and journal information. Discrepancies between review authors were resolved via consensus or by a third author when required. Information extracted from eligible studies included: study eligibility and design; date of publication; country and demographic characteristics of participants; number of experimental conditions; characteristics of employed implementation strategies; study outcomes of interest and information to allow the assessment of risk of bias. Implementation strategies were classified according to the Effective Practice and Organization of Care (EPOC) taxonomy [32] (Appendix III).

#### Assessment of risk of bias in included studies

Two authors independently assessed risk of bias within each included study using the “Risk of Bias” tool described within the *Cochrane Handbook for Systematic Reviews of Interventions* [33]. The following domains were assessed for individual studies and outcomes to determine an overall risk of bias: sequence generation, allocation concealment, blinding of participants and personnel, blinding of outcome assessment, incomplete outcome data, selective outcome reporting, and “other” potential sources of bias. For nonrandomized trials, an additional “potential confounding” domain was also assessed, defined as the risk that an unmeasured characteristic shared by those allocated to receive the implementation intervention (or strategy), rather than the intervention itself, was responsible for reported outcomes [34]. Additional domains were also used to assess cluster-randomized controlled trials, including: recruitment to cluster, baseline imbalance, loss of clusters, incorrect analysis, and compatibility with individually randomized controlled trials [33]. Disagreements between review authors were resolved via consensus or, when required, by a third author.

#### Measurement of treatment effect

Substantial study heterogeneity in outcomes and measures used to assess implementation precluded meta‐analysis and presented considerable synthesis challenges. As such, a narrative synthesis was conducted collectively with studies from the original and updated review. First, we summarized the characteristics of included studies based on population, and “intervention” (implementation strategy categorized based on EPOC taxonomy [32]) characteristics. Second, to provide a high-level summary of findings, we described the effect size of the primary policy, practice, or program implementation outcome measure for each study and summarized this across studies for each broad category of implementation outcomes (e.g., score-based measures, proportion of time implementing a practice, or frequency of implementation) [25, 35]. Effect sizes were calculated by subtracting the change from baseline on the primary implementation outcome for the control (or comparison) group from the change from baseline in the experimental or intervention group. We reverse-scored implementation measures that did not represent an improvement (e.g., proportion of schools serving unhealthy food items) [25, 35]. For studies with multiple follow‐up periods, data from the final follow‐up period reported was extracted and subtracted from baseline [25, 35, 36]. If data to enable the calculation of change from baseline were unavailable, the differences between groups postintervention were used. Where there were two or more primary implementation outcome measures, standardized measures of effect size were calculated for each outcome, measures were ranked based on their size of effect, and the median measure was used (and range reported) [35, 36].

Where the primary outcome measure was not identified by the study authors in the published manuscripts, the implementation outcome on which the study sample size calculation was based was used or, in its absence, the median effect size of all measures judged to be implementation outcomes reported in a manuscript was calculated and the range reported [25, 35, 36]. The inclusion of such effect sizes is for descriptive purposes and should not be considered as pooled estimates of effect as they do not weigh study effects by the inverse of their variance, nor do they consider study issues of study quality or design. Finally, we present a narrative synthesis of all studies, followed by a narrative synthesis of individual studies by the risk factor (physical activity or diet) targeted by the intervention.

## RESULTS

The updated search (August 31, 2016 to April 10, 2019) identified 3,815 unique records of which 62 full-text records and one unpublished study (identified via the trial registry search, findings have since been published [37]) were assessed for eligibility (see Fig. 1). Fifty-five records were excluded following full-text screening for the following reasons: wrong participants (*n* = 1); wrong intervention (*n* = 1); no comparator (*n* = 2); and inappropriate outcomes (*n* = 34). Studies were excluded based on “inappropriate outcomes” if it did not measure implementation of a policy, practice, or program.

In this review update, seven new published studies [26–28, 38–42] and one unpublished study were identified for inclusion (see Fig. 1). Of the 27 studies included in the original Cochrane review covering multiple health risks, 22 studies [43–64] examining healthy eating, physical activity, or obesity prevention policy or practice implemented were included in this review update (see Fig. 1 for reasons for exclusion). In total, 30 studies were included in this review update. See Appendix I for characteristics of included studies.

### Types of studies

Collectively from the 30 studies included from the original and updated review, 19 were conducted in the USA, 7 in Australia, 2 in Canada, and 1 each in New Zealand and the Netherlands. Nineteen included studies employed randomized designs (including 15 cluster randomized) and the remaining 11 studies were nonrandomized with a parallel control group. Studies were conducted between 1985 [59] and 2018 [65], with the duration of the studies varying from 20 weeks [26] to 4 years [49]. Twenty-six of the 30 included studies compared an implementation strategy to a control group or usual practice, whilst the remaining four studies directly compared different implementation strategies [39, 40, 46, 49].

### Participants

The number of schools participating in the studies included in the review varied. The largest study recruited 828 schools [52], whilst the smallest study recruited two schools. The majority of studies (*n* = 22) were conducted within primary (or elementary) schools, which cater for children aged 5–12 years. The remaining studies were conducted in middle schools (*n* = 5) catering for children aged 11–14 years and secondary schools (*n* = 3) catering for children aged 13–18 years. All included studies were conducted within high-income countries.

### Interventions

Sixteen studies tested strategies to implement healthy eating policies, practices, or programs, 11 tested strategies targeting physical activity policies or practices, and 3 tested strategies targeting both healthy eating and physical activity. A comprehensive description of the existing studies in the Cochrane Review are available in the “Characteristics of Included Studies” table of the manuscript [25], whilst a summary of all included 30 studies is provided in Appendix I.

All studies examined multistrategy implementation interventions. The number of implementation strategies, as characterized by the EPOC Taxonomy [32] (see Appendix III), ranged from two to nine (mean number of strategies = 6.5). While there was considerable heterogeneity in the strategies tested, 21 studies tested educational materials and educational meetings in combination with other strategies. Of those other strategies tested, educational outreach visits or academic detailing (*n* = 10) and audit with feedback (*n* = 4) were the most common. No study tested the effectiveness of just one implementation strategy and only two studies [26, 38] tested the same combination of strategies. A summary of the implementation strategies and effects of all included studies is provided in Appendix I.

### Assessment of risk of bias of included studies

**Fig 2:**
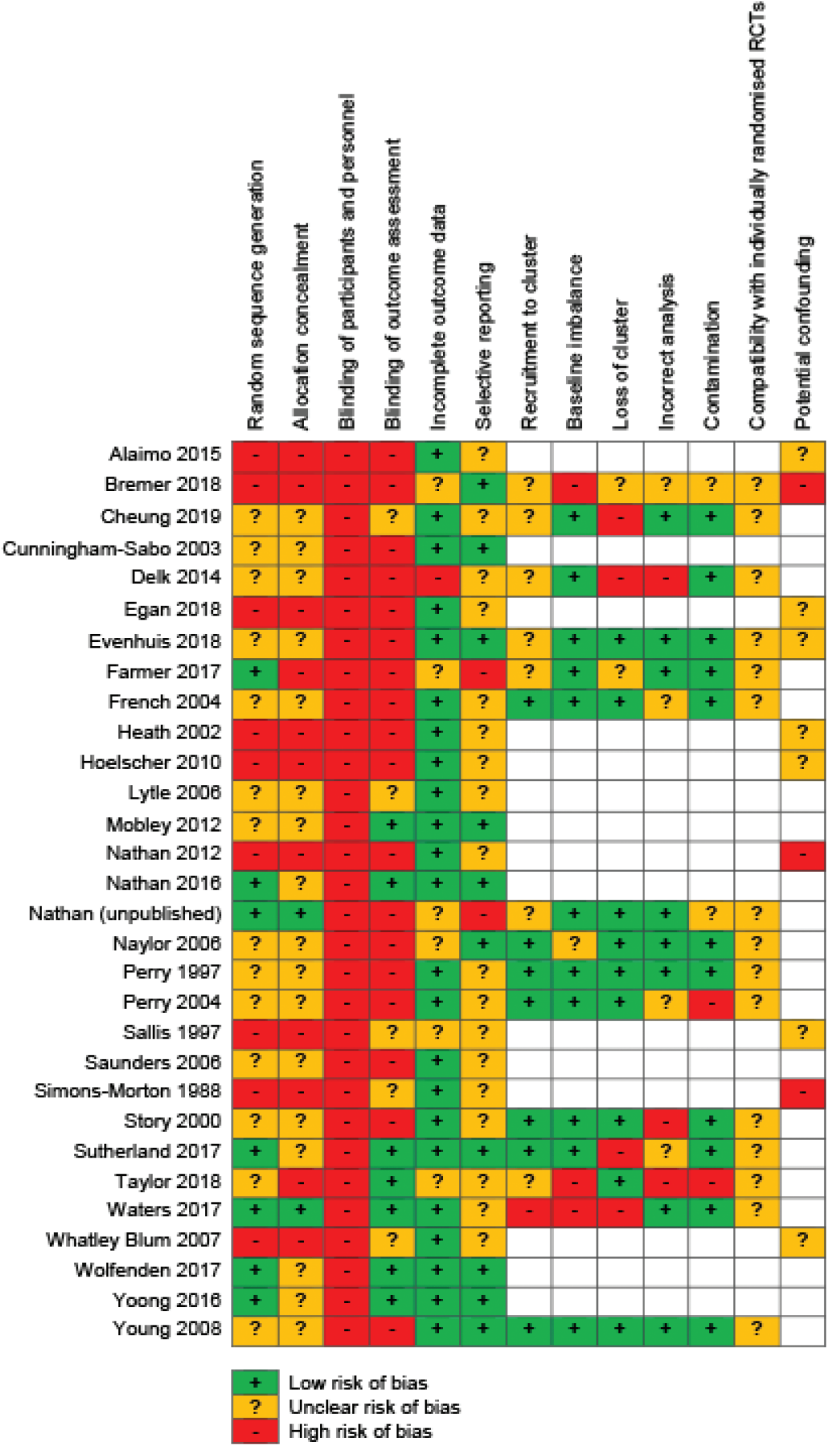
Risk of bias summary.

The “Risk of Bias” assessment for the included studies for each domain is presented in Fig. 2 and described below.

#### Allocation

Risk of bias varied across studies. Nine studies, including eight with nonrandomized designs, were assessed as high risk of selection bias [26, 39, 43, 48, 49, 51, 52, 57, 61]. Thirteen studies, including four RCTs were assessed as unclear risk of selection bias as methods of sequence generation and allocation were not reported [44, 46, 47, 50, 51, 54–56, 58, 64, 66, 67]. While four studies were assessed as low risk of bias for random sequence generation, the method of allocation concealment was unclear [53, 60, 62, 63]. Two studies were assessed as low risk for both sequence generation and allocation concealment [27, 37].

#### Blinding

All studies were assessed as high risk of performance bias as study participants and personnel (e.g., school staff) were not blinded to group allocation. Detection bias varied across studies depending on whether implementation was assessed via self-report (high risk) or objective measures (low risk). Seventeen of the included studies were assessed as high risk [26, 28, 37, 41, 43, 44, 46–49, 52, 54–56, 58, 66, 67] and seven studies were assessed as low risk of detection bias [27, 42, 51, 53, 60, 62, 63]. The remaining five studies were assessed as unclear risk of detection bias due to insufficient information regarding the blinding of data collection staff provided [38, 50, 57, 59, 61].

#### Incomplete outcome data

The majority of studies (*n* = 23) were assessed as low risk of bias as either all or most participating schools were present at follow-up and/or sensitivity analyses were conducted to assess the impact of missing data. The risk of attrition was assessed as unclear in six studies, as insufficient information regarding the loss of schools and treatment of missing data were provided [26, 37, 41, 42, 54, 57]. One study was assessed as high risk of attrition due to untreated missing data at follow-up [46].

#### Selective reporting

Ten studies were assessed as low risk of selective reporting as a trial registration or a published protocol paper was identified and all a priori determined outcomes were reported [26, 44, 51, 53, 54, 60, 62–64, 67]. Two studies were classified as high risk for selective reporting as the implementation outcome was not previously registered in the available protocol or trial registration [37, 41]. For the remaining studies (*n* = 18), the risk of reporting bias was deemed unclear as a published protocol or trial registration was not identified.

#### Other potential sources of bias

For studies using a cluster-RCT design (*n* = 15), additional risk of bias domains were assessed. Potential risk of recruitment (to cluster) was assessed as low for seven studies as randomization occurred either postrecruitment or postbaseline data collection [47, 54–56, 58, 60, 64]. Seven studies were assessed as unclear [26, 37, 38, 41, 42, 46, 67], whilst the remaining study [27] was assessed as high risk of bias due to randomization occurring prior to recruitment and a lack of blinding of recruiters. For risk of bias due to baseline imbalances, the majority of studies (*n* = 11) were assessed as low risk as studies accounted for imbalances by making adjustments for baseline differences during analyses, stratifying by school characteristics or through random allocation of schools to experimental groups [37, 38, 41, 46, 47, 55, 56, 58, 60, 64, 67]. Three studies were assessed as high risk [26, 27, 42], while the remaining study [54] was at unclear risk of bias due to baseline imbalance. Four studies were assessed as high risk for loss of clusters [27, 38, 46, 60]. Three studies were high risk of bias due to incorrect analysis [42, 46, 58], while eight studies were assessed as low risk [27, 37, 38, 41, 54, 56, 64, 67] and the remaining studies (*n* = 4) at unclear risk [26, 47, 55, 60]. Risk of contamination was assessed as low for the majority of clustered studies (*n* = 11), with only two studies assessed as high [42, 55] and the remaining two studies unclear [26, 37].

The potential of confounding factors was assessed as an additional risk of bias domain for nonrandomized trial designs. Of the seven nonrandomized studies, three were considered as high risk of confounding [26, 52, 59], while it was unclear in the remaining five studies whether confounders had been sufficiently adjusted for [39, 43, 57, 61, 67].

### Overall effect of implementation support on policy, practice, or program implementation

Of the 30 included studies, 19 reported significant improvements in at least one implementation outcome (including the one unpublished study) [44, 46–50, 52–58, 60–62, 64, 68]; 3 studies did not report any significant improvements in implementation [26, 43, 63] and 8 did not report any significance tests on such outcomes [27, 38, 39, 42, 51, 59, 66, 67].

Among 10 studies reporting dichotomous implementation strategy outcomes—the proportion of schools or school staff (e.g., classes) implementing a targeted policy, practice, or program—versus a minimal or usual practice control, the median unadjusted (improvement) effect size was 16.2% and ranged from –0.2% to 66.6% [27, 41, 50–53, 60, 62–64].

Six studies reported the percentage of an intervention program or program content that had been implemented, the effects of which were mixed [47, 55, 56, 58, 60, 61]. The unadjusted median effect, relative to the control in the proportion of program or program content implemented, was 23.65% (range –8% to 43%) [47, 55, 56, 58, 60, 61].

Four studies reported the impact of implementation strategies on the time per week that teachers spent implementing physical activity or physical education (PE) lessons, with improvements, relative to control ranging from 5.7 to 54.9 min per week (median = 36.6 min per week; including the one unpublished study) [38, 54, 57].

Among studies reporting other continuous implementation outcomes (e.g., quantity of physical activity lessons), findings were mixed [43, 44, 46, 48, 49, 59, 64, 66]. For example, across the three studies assessing the availability of fruit and vegetables within schools, the median effect size was 1.15 and ranged from 0.64 to 1.23 [42, 55, 58].

Substantial variability in the type of implementation strategies employed in the included studies prevented the impact of specific implementation support strategies, or combinations thereof, from being examined. However, most studies included educational meetings, educational materials in addition to other strategies. The effectiveness of such strategies to achieve improvements in measures of implementation were mixed.

### Implementation of healthy eating policies, practices, and programs

Nineteen of the 30 included studies targeted the implementation of healthy eating practices (13 studies in primary, 4 in middle, and 2 in secondary schools). Studies to improve the implementation of healthy eating policies and practices were dominated by studies to improve the nutritional content or availability of healthy foods as part of U.S. school food services (*n* = 13). In general, such studies reported small improvements in food provision. For example, Cunningham et al. reported reductions in the percentage of energy from fat provided at school breakfast and lunch from –3.3% to –2.7% [44]. Percentage of fat in school meals was reported as reduced by up to 4% in the study by Heath and Coleman [48]. Similarly, in the study by Perry et al., modest although significant reductions were reported in the percentage of kilocalories from fat (–4.3%) and milligrams of sodium (–100.5) in school lunches [56].

Significant improvements were also reported across a range of measures of the percentage of food and beverage items meeting nutrient and portion criteria in a study by Whatley Blum et al. [61]. U.S. studies targeting improvements in the availability of fruits and vegetables in à la carte lines typically significantly increased the mean number of fruit and vegetables options available by between 0.5 to 1.37 [58] or the proportion of schools selling such foods by between 4% and 12% [50]. There was consistent evidence of large effects from Australian randomized trials demonstrating improvement in the availability of healthy foods at school canteens [53, 62, 63]. Three trials demonstrated a dose–response relationship between the intensity of implementation support and school compliance with canteen policies. In the trial by Wolfenden et al., assessing the most intensive implementation strategy—comprised of nine implementation strategies—more than 70% of schools that received multicomponent implementation support (vs. 3% in the control) did not regularly sell foods that were restricted or banned from sale by healthy canteen guidelines, and more than 80% (versus 27% in the control) had more than half of all foods for sale as healthy (“green”) products [62]. An Australian study also reported significant improvement relative to control (16%) in the implementation of fruit and vegetable breaks during class time [52]. Large changes were also reported in a small randomized trial (12 schools per group), in the presence of a written school nutrition or policy, but not canteen policy, in a trial by Waters et al. [27].

### Implementation of physical activity policies, practices, and programs

Fourteen of the 30 included studies targeted the implementation of physical activity policies and practices (nine studies in primary, four in middle, and one in secondary school). Studies testing strategies to improve the implementation of physical activity policies and practices focused on measures of time that classroom teachers spent delivering PE or structured physical activity each week, the quality of PE lessons (e.g., lesson time allocated to children engaging in physical activity), or the implementation of specific elements of physical activity interventions [26, 27, 38, 39, 41, 46, 49, 54, 56, 57, 60, 64, 66]. Studies targeting the time spent on PE typically saw significant improvements following multistrategy implementation support [38, 54, 57]. For example, in their Canadian study, Naylor et al. reported significant improvements in classroom time spent on PE relative to control of up to 1 hr per week [54]. Similarly, one study by Nathan et al. found significant improvements in the minutes per day that teachers scheduled physical activity relative to control [37]. Sallis et al. found significant increases in the duration per week of PE lessons relative to control of 26.6 min and significant increases in the frequency of PE lessons a week [57]. However, Cheung et al. found far smaller changes in the mean minutes of physical activity offered per week, ranging from −2.4 to 13 min (significance not reported) [38].

Three studies compared implementation strategies to a usual care or minimal support control on measures of lesson quality [26, 56, 60]. Perry et al. reported a significant increase of 14% relative to control, in the proportion of quality activities observed, relative to control in PE lessons following implementation support [56]. Significant improvements were also reported in physical activity program quality score in an Australian randomized trial by Sutherland et al. [60] but not in measures of quality of PE lessons in a more recent study by Bremer et al. [26] among schools receiving implementation support. Among studies that assessed changes in the implementation of a physical activity policy, practice, or program [27, 41, 60, 64], effects were modest with median effect sizes ranging from no change (–0.2%) in the study by Farmer et al. [41] to a change of almost 20% in the Australian randomized trial by Sutherland et al. [60].

## DISCUSSION

This review aimed to examine the impact of strategies to improve the implementation of policies, practices, and programs to promote healthy eating, physical activity, or prevent obesity within school-based settings. Despite the substantial number of efficacious school-based behavioral interventions published in the last 10 years [13, 69], and the increase in implementation science research during the same period, this review only identified an additional eight studies since the publication of the original review in 2017 [25]. Collectively, from the newly included studies and the 22 studies included in the original review by Wolfenden et al. [25], most studies employed randomized controlled trial designs to test multicomponent implementation support strategies. Despite considerable heterogeneity in the effects of implementation strategies, the findings provide some evidence to support the effectiveness of strategies in enhancing the nutritional quality of foods served at schools [61], implementation of canteen nutrition policies [62], improvements in the time scheduled for PE [57], and the quality of PE lessons [54]. Such evidence could provide some guidance for school-based settings and jurisdictions seeking to implement recommendations within the WHO Commission on Ending Childhood Obesity Report [11] and the WHO Health Promoting Schools Framework [70].

The median effect size of the primary implementation outcomes reported in this review (16.5%, range 0.2% to 66.6%) are comparable with implementation efforts in other community settings. For example, in a recent review of implementation strategies to improve healthy eating and physical activity promoting policies and practices in childcare, the median effect size in the proportion of staff implementing a policy or practice was 11% (range 2.5 to 33%) [35]. Similarly, in a review of strategies to improve healthy eating and physical activity policies and practices in workplaces, the median effect size in the proportion of workplaces implementing a policy or practice was 13.4% (range 10.9%–39.6%) [36]. Effect sizes and range of effects reported across these reviews suggests that implementation strategies typically yield modest but highly variable improvements in implementation. Such findings indicate that while it is possible to result in large improvements (up to 66.6% in this instance) in the implementation of policies and practices, the effects of implementation strategies are likely dependent on context, the factors impeding implementation, and the extent to which the selected implementation strategies adequately address these. Research to better identify the most potent mix of implementation supports given barriers and context, therefore, should be a priority for future research in the field.

While limited, there is an accumulating body of evidence to suggest that implementation strategies have resulted in small improvements in the availability and provision of healthy foods in schools [40, 44, 52]. Given that food consumed at school contributes to an estimated 40% of a child’s daily energy intake [71], small improvements in consumption could lead to important improvement in public health nutrition. For example, studies modeling the impact of reductions in energy intake have found a small decrease in energy intake of 290 kJ per day could be sufficient in preventing excessive weight gain in children [72]. Several studies within the review found significant reductions in energy from fat [44, 48, 56] and total energy [59] provided by lunch meals, in magnitudes exceeding 200 kJ, that could make a meaningful contribution to such reductions. The potential benefit of such improvements, however, is maximized when implementation occurs at scale. Disappointingly, just 5 of the 30 studies included in this review (including three targeting implementation of nutrition interventions) examined the impact of implementation occurring “at scale,” defined as 50 or more schools [37, 43, 52, 56, 73]. Effects were mixed among these studies, with three reporting significant improvements in the majority of implementation outcomes [37, 52, 56], whilst the remaining two studies reporting no improvement [38, 43]. As the effects of interventions may attenuate when delivered on a larger scale, the potential benefit of strategies to improve population-wide implementation of school health initiatives remains uncertain.

## LIMITATIONS

Substantial variation across included studies in the implementation strategies employed, policies, practices, and programs targeted and measures used to assess implementation resulted in considerable challenges during data synthesis and interpretation of findings. Studies tested a range of multicomponent implementation strategies, with only two studies testing the same combination [26, 38]. As such, the impact of specific implementation support strategies or combinations thereof were unable to be examined. In contrast to a similar review conducted within the childcare setting [35] where a number of studies used similar score-based measures of implementation, enabling the pooling of studies for meta-analysis, such homogeneity in outcomes and measures was not evident within this review. Due to this, synthesizing the data and drawing comparisons between outcomes within this review was difficult. Additionally, with 18 studies recruiting a sample of less than 30 schools, 19 studies using nonvalidated self-reported measures of implementation, and all but 2 studies assessed as high risk of bias in multiple domains, the true improvements in policy and practice implementation may be unable to be detected. Finally, a lack of consistent terminology and inadequate reporting of employed implementation strategies across studies is an important limitation of this review.

Despite best efforts from the authors, the review process was not without its limitations. A search of international implementation journals and a hand search of reference lists of included studies was not conducted, which may have identified additional eligible studies to contribute to the findings of this review. Finally, the review extracted a limited range of the many trial characteristics, outcomes, and other structural or contextual factors that may influence implementation. Greater extraction and reporting of a broader range of such characteristics would improve the external validity and utility of the findings by the end user. As such, future reviews should consider coding and reporting studies using the Reach, Effectiveness, Adoption, Implementation and Maintenance (RE-AIM) framework [74, 75].

## CONCLUSION

Despite the field of implementation science rapidly evolving and a considerable amount of research being conducted in the schools setting, a lack of strong and consistent evidence remains to support the selection of strategies to improve the implementation of healthy eating, physical activity, and obesity-prevention policies, practices, and programs. In the absence of clear evidence from empirical studies, researchers, and practitioners responsible for health promotion in school-based settings may have to rely on considerable formative research (e.g., consultation with schools to identify barriers and enablers) and theory to guide implementation. Future research calls for studies of high methodological quality using validated and consistent measures of implementation whilst adequately reporting employed implementation strategies using taxonomies, such as the EPOC [32] or Expert Recommendations for Implementing Change [76] taxonomies.

## Supplementary Material

ibab037_suppl_Supplementary_Material

## Notes

**Funding:** We would like to acknowledge the funding support provided by New South Wales Office Preventive Health. C.B. is supported by a cofunded industry scholarship between Hunter New England Population Health and the University of Newcastle; N.N. is supported by a Hunter New England Clinical Research Fellowship; S.Y. is funded by an Australian Research Council Discovery Early Career Researcher Award (DECRA) (DE170100382); R.S. is supported by a National Health and Medical Research Council Translating Research into Practice Fellowship (APP1150661); L.W. is a Hunter New England Clinical Research Fellow and is supported by Heart Foundation Future Leader Fellowship (Award No. 101175) and a National Health and Medical Research Council Career Development Fellowship (APP1128348).

## Appendix A1

**Table:** Summary of intervention, measures, and absolute intervention effect size in included studies

Study	Targeted risk factor	Implementation strategies	Comparison	Primary implementation outcome and measures	Effect size	Number of measures with significant result (*p* < .05) favoring the intervention
Alaimo et al. [43]	N	Clinical practice guidelines, educational materials, educational outreach visits or academic detailing, external funding, local consensus processes, tailored interventions	Usual practice or waitlist control	Continuous: Nutrition policy score and nutrition education and/or practice score (two measures)	Median (range): 0.65 (0.2 to 1.1)	0/2
Cunningham- Sabo et al. [44]	N	Clinical practice guidelines, educational materials, educational meetings, educational outreach visits or academic detailing	Usual practice	Continuous: Nutrient content of school meals % of calories from fat breakfast/lunch (two measures)	Median (range): −3% (−3.3% to −2.7%)	1/2
Delk et al. [46]	PA	Local consensus process, educational meetings, clinical practice guidelines, educational outreach visits or academic detailing, tailored interventions, other	Different implementation strategy	Continuous: % of teachers that conducted activity breaks weekly (one measure, two comparisons) Dichotomous: % implementing a variety of policies and practices (two measures, four comparisons)	Median (range): 13.3% (11.1% to 15.4%) Median (range): 26.5% (19.4% to 31.9%)	6/6
French et al. [47]	N	Local consensus processes, tailored intervention, educational meetings, pay for performance	Usual practice or waitlist control	Continuous % of program implementation (five measures)	Median (range): 33% (11% to 41%)	5/5
Heath and Coleman [48]	N	Educational materials, educational meetings, educational outreach visits or academic detailing	Usual practice	Continuous: % fat in school meal (two measures) Sodium of school meals (two measures)	Median (range): −1.7% (−4.4% to 1%) Median (range): −29.5 (−48 to −11)	1/4
Hoelscher et al. [49]	N/PA	Educational materials, educational meetings, educational outreach visits or academic detailing, pay for performance, other, the use of information and communication technology, local consensus process	Different implementation strategy	Continuous: Mean number of lessons/or activities (five measures) Dichotomous: % implementing a variety of policies and practices (two measures)	Median (range): 0.8 (−0.4 to 1. 2) Median (range): 4.4% (3.6% to 5.2%)	4/7
Lytle et al. [50]	N	Educational materials, educational meetings, local opinion leaders, local consensus processes	Usual practice or waitlist control	Dichotomous: % of schools offering or selling targeted foods (four measures)	Median (range): 8.5% (4% to 12%)	2/4
Mobley et al. [51]	N	Educational games, educational meetings, external funding, tailored intervention, educational materials, educational outreach or academic detailing, other, the use of information and communication technology	Usual practice or waitlist control	Dichotomous: % schools meeting various nutrition goals (12 measures)	Median (range): 15.5% (0% to 88%)	NR
Nathan et al. [52]	N	Educational materials, educational meetings, local consensus processes, local opinion leaders, other, monitoring the performance of the delivery of the healthcare, tailored interventions	Minimal support control	Dichotomous: % Schools implementing a vegetable and fruit break (one measure)	Mean difference (95% CI): 16.2% (5.6% to 26.8%)	1/1
Nathan et al. [53]	N	Audit and feedback, continuous quality improvement, education materials, education meeting, local consensus process, local opinion leader, tailored intervention, other	Usual practice	Dichotomous: % implementing a variety of policies and practices (two measures)	Median (range): 35.5% (30.0% to 41.1%)	2/2
Naylor et al. [54]	PA	Educational materials, educational meetings, educational outreach meetings or academic detailing, local consensus process, other, tailored interventions	Usual practice or waitlist control	Continuous: Minutes per week of physical activity implemented in the classroom (one measure, two comparisons)	Median (range): 54.9 min (46.4 to 63.4)	2/2
Perry et al. [56]	N/PA	Educational materials, educational meetings, educational outreach visits or academic detailing, other	Usual practice or waitlist control	Continuous: % of kilocalories from fat in school lunch (one measure) Mean milligrams of sodium in lunches (one measure) Cholesterol milligrams in lunches (one measure) Quality of PE lesson % of seven activities observed (one measure)	Mean difference (95%CI): −4.3% (−5.8% to −2.8%) Mean difference (95% CI): −100.5 (−167.6 to −33.4) Mean difference (95% CI): −8.3 (−16.7 to 0.1) Mean difference (95% CI): 14.3% (11.6% to 17.0%)	3/4
Perry et al. [55]	N	Educational meetings, educational outreach visits or academic detailing, educational materials, local consensus processes, other	Usual practice or waitlist control	Continuous: % of program implementation (two measures) Mean number of fruit and vegetables available (two measures)	Median (range): 14% (−2% to 30%) Median (range): 0.64 (0.48 to 0.80)	2/4
Sallis et al. [57]	PA	Educational materials, educational meetings, educational outreach visits or academic detailing, length of consultation, other	Usual practice or waitlist control	Continuous: Duration (minutes) per week of physical education lessons (one measure) Frequency (per week) of physical education lessons (one measures)	Mean difference (95% CI): 26.6 (15.3 to 37.9) Mean difference (95% CI): 0.8 (0.3 to 1.3)	2/2
Saunders et al. [66]	PA	Educational materials, educational meetings, educational outreach visits or academic detailing, local consensus processes, local opinion leaders, other	Usual practice or waitlist control	Continuous: School level policy and practice related to physical activity (nine measures)	N/A	NR
Simons- Morton et al. [59]	N	Educational materials, educational outreach visits or academic detailing, local consensus processes, local opinion leaders, managerial supervision, monitoring of performance, other	Usual practice	Continuous: Macronutrient content of school meals (two measures)	N/A	NR
Story et al. [58]	N	Educational meetings, other	Usual practice	Continuous: Mean number of fruit and vegetables available (two measures) % of guidelines implemented and % of promotions held (four measures)	Median (range): 1.15 (1 to 1.3) Median (range): 38.4% (28.5% to 43.8%)	6/6
Sutherland et al. [60]	PA	Audit and feedback, education materials, education meeting, education outreach visits or academic detailing, local opinion leader, other	Usual practice or waitlist control	Dichotomous: % implementing a variety of policies and practices (two measures) Continuous: Physical education lesson quality score (one measures) % of program implementation (four measures)	Median (range): 19% (16% to 22%) Mean difference: 21.5 Median (range): −8% (−18% to 2%)	0/2 1/1 0/4
Whatley Blum et al. [61]	N	Clinical practice guidelines, educational materials, educational meetings, educational outreach visits or academic detailing, external funding, distribution of supplies, local consensus process, other	Usual practice or waitlist control	Continuous: % of food and beverage items meeting guideline nutrient and portion criteria (six measures)	Median (range): 42.95% (15.7% to 60.6%)	5/6
Wolfenden et al. [62]	N	Audit and feedback, continuous quality improvement, external funding, education materials, education meeting, education outreach visits or academic detailing, local consensus process, local opinion leader, tailored intervention	Usual practice	Dichotomous: % implementing a variety of policies and practices (two measures)	Median (range): 66.6% (60.5% to 72.6%)	2/2
Yoong et al. [63]	N	Audit and feedback, continuous quality improvement, education materials, tailored intervention	Usual practice	Dichotomous: % implementing a variety of policies and practices (two measures)	Median (range): 21.6% (15.6% to 27.5%)	0/2
Young et al. [64]	PA	Education materials, education meetings, educational outreach visits or academic detailing, interprofessional education, local consensus processes, local opinion leaders	Usual practice	Dichotomous: % implementing a variety of policies and practices (seven measures) Continuous: Average number of physical activity programs taught (one measure)	Median (range): 9.3% (−6.8% to 55.5%) Effect size (95% CI): 5.1 (−0.4 to 10.6)	1/8
New studies identified in this review						
Bremer et al. [26]	PA	Educational meetings, educational materials	Usual practice	Continuous: Quantity of physical education lessons (one measure)	Mean difference: *t*(27) = −0.23,	0/1
Cheung et al. [38]	PA	Educational meeting, educational materials	Usual practice	Continuous: Mean minutes of physical activity offered per week (three measures)	Median (range): 5.7 (−2.4 to 13)	NR
Egan et al. [39]	PA	Educational materials; educational outreach visit or academic detailing, tailored intervention, audit, and feedback	Alternate intervention or usual practice	Continuous:Mean implementation score for components of movement integration (five measures)	Median (range): −2.79 (−4.92 to 3.66)	NR
Evenhuis et al. [40]	N	Educational materials, educational meeting, audit with feedback, educational outreach visit or academic detailing	Educational materials	Continuous: Availability of healthier food products on display (one measure) Healthier product accessibility (one measure)	Mean difference: 16.79 Mean difference: 9	NR
Farmer et al. [41]	PA	Incentives, local consensus approach, tailored interventions	Usual practice	Dichotomous: % implementing a variety of policies and practices (one measure) Continuous: Provision of play opportunities (one measure)	Mean difference (95% CI): −0.20 (−11.46 to 11.06) Mean difference (95% CI): 4.50 (1.82 to 7.18)	0/1 1/1
Nathan, unpublished data	PA	Educational outreach visits, centralized technical support, mandate change, identify and prepare champions, provide ongoing consultation, educational material	Usual practice	Continuous: Mean minutes of teacher’s scheduled PA per day	Mean difference (95% CI): 36.60 (2.68 to 70.51)	1/1
Taylor et al. [42]	N	Incentives, educational materials, educational outreach visits, or academic detailing	Usual practice or waitlist control	Continuous: Quantity of fruit and vegetables available (two measures)	Median (range): 1.23 (−0.79 to 3.26)	NR
Waters et al. [27]	N/PA	Educational materials, educational outreach visits or academic detailing; local consensus approach, tailored interventions	Usual practice	Dichotomous: % implementing a variety of policies and practices (three measures)	Median (range): 7% (−11.7% to 15%)	NR

*CI* confidence interval; *N* nutrition; *NR* not reported; *PA* physical activity.

## Appendix A2

**Table:** Search StrategyDatabase(s): Ovid MEDLINE(R) and Epub ahead of print, in-process and other nonindexed citations and daily 1946 to April 10, 2019 Search strategy:

#	Searches	Results
1	schools/	34,641
2	((primary or elementary or middle or junior or high or secondary) adj (school* or student*)).mp.	61,499
3	kinder*.mp.	22,544
4	1 or 2 or 3	106,292
5	implement*.tw.	427,155
6	Health Promotion/mt [Methods]	19,229
7	“Outcome and Process Assessment (Health Care)”/	25,691
8	“Process Assessment (Health Care)”/	4,389
9	“Outcome Assessment (Health Care)”/	67,208
10	Program Evaluation/	59,115
11	Interrupted Time Series Analysis/	553
12	dissemin*.tw.	115,236
13	adopt*.tw.	220,568
14	practice.tw.	634,669
15	organi?ational change*.tw.	2,613
16	diffus*.tw.	353,856
17	(system* adj2 change*).tw.	15,325
18	quality improvement*.tw.	30,437
19	transform*.tw.	452,648
20	translat*.tw.	283,791
21	transfer*.tw.	594,534
22	uptake*.tw.	335,586
23	sustainab*.tw.	55,964
24	institutionali*.tw.	14,726
25	routin*.tw.	355,436
26	maintenance.tw.	254,465
27	capacity.tw.	460,913
28	incorporat*.tw.	395,520
29	adher*.tw.	172,945
30	((polic* or practice* or program* or innovation*) adj5 (performance or feedback or prompt* or reminder* or incentive* or penalt* or communicat* or social market* or professional development or network* or leadership or opinion leader* or consensus process* or change manage* or train* or audit*)).tw.	103,076
31	integrat*.tw.	460,724
32	scal* up.tw.	16,615
33	5 or 6 or 7 or 8 or 9 or 10 or 11 or 12 or 13 or 14 or 15 or 16 or 17 or 18 or 19 or 20 or 21 or 22 or 23 or 24 or 25 or 26 or 27 or 28 or 29 or 30 or 31 or 32	4,833,658
34	exp Obesity/	195,8011
35	Weight Gain/	29,698
36	exp Weight Loss/	38,540
37	obes*.tw.	269,101
38	(weight gain or weight loss).tw.	130,488
39	(overweight or over weight or overeat* or over eat*).tw.	64,358
40	weight change*.tw.	10,275
41	((bmi or body mass index) adj2 (gain or loss or change)).tw.	4,130
42	exp Primary Prevention/	143,568
43	(primary prevention or secondary prevention).tw.	30,738
44	(preventive measure* or preventative measure*).tw.	22,909
45	(preventive care or preventative care).tw.	5,038
46	(obes* adj2 (prevent* or treat*)).tw.	19,978
47	34 or 35 or 36 or 37 or 38 or 39 or 40 or 41 or 42 or 43 or 44 or 45 or 46	633,369
48	exp Exercise/	176,978
49	physical activity.tw.	94,644
50	physical inactivity.tw.	6,883
51	Motor Activity/	94,188
52	(“physical education” or “physical training”).tw.	9,495
53	“Physical Education and Training”/	13,213
54	Physical Fitness/	26,208
55	sedentary.tw.	27,694
56	exp Life Style/	85,835
57	exp Leisure Activities/	220,470
58	Dancing/	2,669
59	dancing.tw.	1,576
60	(exercise* adj aerobic*).tw.	186
61	sport*.tw.	66,644
62	((lifestyle* or life style*) adj5 activ*).tw.	6,082
63	48 or 49 or 50 or 51 or 52 or 53 or 54 or 55 or 56 or 57 or 58 or 59 or 60 or 61 or 62	547,175
64	exp Diet/	261,598
65	nutrition*.tw.	248,427
66	healthy eating.tw.	5,898
67	Child Nutrition Sciences/	1,075
68	fruit*.tw.	95,658
69	vegetable*.tw.	49,588
70	“Fruit and Vegetable Juices”/	1,248
71	canteen*.tw.	589
72	food service*.tw.	1,810
73	menu*.tw.	4,561
74	calorie*.tw.	24,033
75	Energy Intake/	38,728
76	energy density.tw.	8,494
77	Eating/	50,500
78	Feeding Behavior/ or feeding behavio?r*.tw.	81,927
79	dietary intake.tw.	21,918
80	Food Habits/	77,114
81	Food/	31,390
82	Carbonated Beverages/ or soft drink*.mp.	5,116
83	soda.tw.	3,799
84	sweetened drink*.tw.	262
85	Dietary Fats, Unsaturated/ or Dietary Fats/	51,350
86	confectionar*.tw.	240
87	(school adj (lunch* or meal*)).tw.	1,439
88	menu plan*.tw.	184
89	((feeding or food or nutrition*) adj program*).tw.	4,133
90	cafeteria*.tw.	1,848
91	Nutritional Status/	40,791
92	64 or 65 or 66 or 67 or 68 or 69 or 70 or 71 or 72 or 73 or 74 or 75 or 76 or 77 or 78 or 79 or 80 or 81 or 82 or 83 or 84 or 85 or 86 or 87 or 88 or 89 or 90 or 91	741,173
93	exp Smoking/	140,042
94	exp “Tobacco Use Cessation”/	1,064
95	smok*.tw.	258,516
96	Nicotine/	24,526
97	Tobacco/ or “Tobacco Use”/	30,560
98	((ceas* or cess* or prevent* or stop* or quit* or abstin* or abstain* or reduc*) adj5 (smok* or tobacco or nicotine)).tw.	51,511
99	“Tobacco Use Disorder”/	10,617
100	ex-smoker*.tw.	3,769
101	anti-smok*.tw.	1,225
102	93 or 94 or 95 or 96 or 97 or 98 or 99 or 100 or 101	335,144
103	alcohol drinking/ or binge drinking/	64,411
104	alcohol*.tw.	308,065
105	Alcoholic Intoxication/ or Alcoholism/	82,939
106	drink*.tw.	128,749
107	liquor*.tw.	7,780
108	beer*.tw.	9,611
109	wine*.tw.	18,647
110	spirit*.tw.	24,880
111	drunk*.tw.	4,203
112	intoxicat*.tw.	44,075
113	binge.tw.	11,829
114	103 or 104 or 105 or 106 or 107 or 108 or 109 or 110 or 111 or 112 or 113	508,479
115	47 or 63 or 92 or 102 or 114	2,374,155
116	Randomized Controlled Trial/	479,844
117	clinical trial/ or controlled clinical trial/	537,645
118	random allocation/	98,475
119	Double-Blind Method/	150,664
120	Single-Blind Method/	26,573
121	placebos/	34,301
122	Research Design/	100,656
123	Evaluation Studies/	242,326
124	Comparative Study/	1,826,707
125	exp Longitudinal Studies/	1,22,430
126	Cross-Over Studies/	45,007
127	exp Cohort studies/	1,844,224
128	Controlled Before-After Studies/	383
129	Interrupted Time Series Analysis/	553
130	comparative study.pt.	1,826,707
131	clinical trial.tw.	125,184
132	latin square.tw.	4,495
133	(time adj series).tw.	26,782
134	(before adj2 after adj3 (stud* or trial* or design*)).tw.	12,708
135	((singl* or doubl* or trebl* or tripl*) adj5 (blind* or mark)).tw.	160,930
136	placebo*.tw.	202,959
137	random*.tw.	1,038,274
138	(matched adj (communit* or school* or population*)).tw.	2,305
139	control*.tw.	3,546,542
140	(comparison group* or control group*).tw.	434,335
141	matched pairs.tw.	5,809
142	outcome stud*.tw.	7,564
143	(qua?iexperimental or qua?i experimental or pseudo experimental).tw.	11,696
144	(nonrandomi?ed or non randomi?ed or psuedo randomi?ed or quasi randomi?ed).tw.	26,473
145	prospectiv*.tw.	638,036
146	volunteer*.tw.	182,708
147	116 or 117 or 118 or 119 or 120 or 121 or 122 or 123 or 124 or 125 or 126 or 127 or 128 or 129 or 130 or 131 or 132 or 133 or 134 or 135 or 136 or 137 or 138 or 139 or 140 or 141 or 142 or 143 or 144 or 145 or 146	7,432,338
148	exp adolescent/ or child/	2,671,427
149	(child or children or adolescen* or teen*).tw.	1,276,835
150	148 or 149	3,119,058
151	4 and 33 and 115 and 147 and 150	4,111
152	limit 151 to ed=20160901-20190412	823

## DATABASE(S): EMBASE 1947 TO PRESENT SEARCH STRATEGY:

**Table:**

#	Searches	Results
1	schools/	63,598
2	((primary or elementary or middle or junior or high or secondary) adj (school* or student*)).mp.	83,026
3	kinder*.mp.	32,382
4	1 or 2 or 3	166,381
5	implement*.tw.	557,138
6	dissemin*.tw.	158,810
7	adopt*.tw.	281,184
8	practice.tw.	870,432
9	organi?ational change*.tw.	3,193
10	diffus*.tw.	478,311
11	system* change*.tw.	8,902
12	quality improvement*.tw.	46,116
13	transform*.tw.	535,815
14	translat*.tw.	350,917
15	transfer*.tw.	714,912
16	uptake*.tw.	442,073
17	sustainab*.tw.	67,481
18	institutionali*.tw.	19,660
19	routin*.tw.	539,589
20	maintenance.tw.	351,213
21	capacity.tw.	602,641
22	incorporat*.tw.	494,048
23	adher*.tw.	252,374
24	((polic* or practice* or program* or innovation*) adj5 (performance or feedback or prompt* or reminder* or incentive* or penalt* or communicat* or social market* or professional development or network* or leadership or opinion leader* or consensus process* or change manage* or train* or audit*)).tw.	141,527
25	integrat*.tw.	561,121
26	scal* up.tw.	20,866
27	health care quality/	231,534
28	quality control/	170,122
29	program evaluation/	12,357
30	total quality management/	55,032
31	5 or 6 or 7 or 8 or 9 or 10 or 11 or 12 or 13 or 14 or 15 or 16 or 17 or 18 or 19 or 20 or 21 or 22 or 23 or 24 or 25 or 26 or 27 or 28 or 29 or 30	6,381,792
32	exp Obesity/	482,160
33	Weight Gain/	91,702
34	Weight Loss.tw. or exp weight reduction/	135,406
35	obes*.tw.	406,493
36	(weight gain or weight loss).tw.	200,827
37	(overweight or over weight or overeat* or over eat*).tw.	97,808
38	weight change*.tw.	15,239
39	((bmi or body mass index) adj2 (gain or loss or change)).tw.	6,986
40	exp Primary Prevention/	37,972
41	(primary prevention or secondary prevention).tw.	46,930
42	(preventive measure* or preventative measure*).tw.	32,787
43	(preventive care or preventative care).tw.	6,298
44	(obes* adj2 (prevent* or treat*)).tw.	28,499
45	32 or 33 or 34 or 35 or 36 or 37 or 38 or 39 or 40 or 41 or 42 or 43 or 44	850,700
46	exp Exercise/	336,319
47	physical activity.tw. or exp physical activity/	433,582
48	physical inactivity.tw.	9,388
49	exp Motor Activity/	536,018
50	(“physical education” or “physical training”).tw.	13,789
51	physical education/	13,316
52	physical fitness.tw. or fitness/	41,592
53	sedentary.tw.	37,170
54	lifestyle/	104,759
55	Leisure Activit*.tw. or leisure/	34,038
56	exp Sports/	157,505
57	Dancing/	4,479
58	(dance* or dancing).tw.	8,367
59	(exercise* adj2 aerobic*).tw.	12,926
60	sport*.tw.	91,539
61	((lifestyle* or life style*) adj5 activ*).tw.	8,789
62	46 or 47 or 48 or 49 or 50 or 51 or 52 or 53 or 54 or 55 or 56 or 57 or 58 or 59 or 60 or 61	1,385,883
63	exp Diet/	340,795
64	nutrition*.tw. or nutrition/	386,929
65	(health* adj2 eat*).tw.	10,407
66	nutritional science/	5,719
67	fruit*.mp. or fruit/ or “fruit and vegetable juice”/	143,342
68	vegetable*.tw. or vegetable/	76,624
69	canteen*.tw.	978
70	Food Services.tw. or catering service/	18,558
71	menu*.tw.	5,961
72	(calorie or calories or kilojoule*).tw.	36,880
73	Energy Intake.tw. or caloric intake/	64,139
74	energy density.tw.	6,365
75	Eating/	35,119
76	Feeding Behavior/ or feeding behavio?r*.tw.	86,486
77	dietary intake.tw. or dietary intake/	86,822
78	Food Habits/	67,209
79	Food/	91,993
80	Carbonated Beverages/ or soft drink*.mp.	6,974
81	soda.tw.	5,149
82	sweetened drink*.tw.	360
83	Dietary Fats, Unsaturated/ or Dietary Fats/	48,641
84	confectionar*.tw.	341
85	(school adj (lunch* or meal*)).tw.	1,836
86	((feeding or food or nutrition*) adj program*).tw.	5,015
87	cafeteria*.tw.	2,308
88	Nutritional Status/	62,601
89	63 or 64 or 65 or 66 or 67 or 68 or 69 or 70 or 71 or 72 or 73 or 74 or 75 or 76 or 77 or 78 or 79 or 80 or 81 or 82 or 83 or 84 or 85 or 86 or 87 or 88	1,089,562
90	exp Smoking/	368,685
91	exp “Tobacco Use Cessation”/	54,469
92	smok*.tw.	382,866
93	Nicotine/	47,085
94	Tobacco/ or “Tobacco Use”/	54,004
95	((ceas* or cess* or prevent* or stop* or quit* or abstin* or abstain* or reduc*) adj5 (smok* or tobacco or nicotine)).tw.	66,147
96	“Tobacco Use Disorder”/	7,394
97	ex-smoker*.tw.	6,694
98	anti-smok*.tw.	1,588
99	90 or 91 or 92 or 93 or 94 or 95 or 96 or 97 or 98	545,883
100	alcohol drinking/ or binge drinking/	40,705
101	alcohol*.tw.	442,943
102	Alcoholic Intoxication/ or Alcoholism/	133,150
103	drink*.tw.	178,445
104	liquor*.tw.	12,281
105	beer*.tw.	13,964
106	wine*.tw.	23,346
107	spirit*.tw.	32,568
108	drunk*.tw.	6,026
109	intoxicat*.tw.	66,687
110	binge.tw.	16,608
111	100 or 101 or 102 or 103 or 104 or 105 or 106 or 107 or 108 or 109 or 110	716,291
112	45 or 62 or 89 or 99 or 111	3,879,229
113	Randomized Controlled Trial/	544,426
114	clinical trial/ or controlled clinical trial/	1,037,260
115	random allocation/	78,229
116	Double-Blind Method/	126,778
117	Single-Blind Method/	32,642
118	placebos/	285,441
119	Research Design/	1,626,031
120	Intervention Studies/	31,912
121	Evaluation Studies/	38,259
122	Comparative Study/	835,481
123	exp Longitudinal Studies/	124,566
124	Cross-Over Studies/	48,369
125	clinical trial.tw.	183,772
126	latin square.tw.	4,848
127	(time adj series).tw.	30,180
128	(before adj2 after adj3 (stud* or trial* or design*)).tw.	17,774
129	((singl* or doubl* or trebl* or tripl*) adj5 (blind* or mark)).tw.	226,356
130	placebo*.tw.	291,972
131	random*.tw.	1,404,881
132	(matched adj (communit* or school* or population*)).tw.	3,301
133	control*.tw.	4,744,140
134	(qua?iexperimental or qua?i experimental or pseudo experimental).tw.	14,109
135	(nonrandomi?ed or non randomi?ed or psuedo randomi?ed or quasi randomi?ed).tw.	35,465
136	prospectiv*.tw.	970,850
137	volunteer*.tw.	248,636
138	cohort analysis/ or cohort studies/	452,474
139	113 or 114 or 115 or 116 or 117 or 118 or 119 or 120 or 121 or 122 or 123 or 124 or 125 or 126 or 127 or 128 or 129 or 130 or 131 or 132 or 133 or 134 or 135 or 136 or 137 or 138	9,238,197
140	school child/	343,648
141	adolescent/	1,559,797
142	(child or children or adolescen* or teen*).tw.	1,768,364
143	140 or 141 or 142	2,887,338
144	4 and 31 and 112 and 139 and 143	4,962
145	limit 144 to dd=20160901-20190412	688

## DATABASE(S): PSYCINFO 1806 TO APRIL WEEK 2 2019 SEARCH STRATEGY:

**Table:**

#	Searches	Results
1	schools/	28,501
2	((primary or elementary or middle or junior or high or secondary) adj (school* or student*)).mp.	187,658
3	kinder*.mp.	25,418
4	1 or 2 or 3	229,493
5	implement*.tw.	162,915
6	Dissemin*.tw.	10,234
7	adopt*.tw.	87,104
8	practice.tw.	316,893
9	organi?ational change*.tw.	7,087
10	diffus*.tw.	28,195
11	system* change*.tw.	3,736
12	quality improvement*.tw.	4,661
13	transform*.tw.	73,504
14	translat*.tw.	53,620
15	transfer*.tw.	69,749
16	uptake*.tw.	14,796
17	sustainab*.tw.	18,291
18	institutionali*.tw.	16,494
19	routin*.tw.	44,817
20	maintenance.tw.	57,427
21	capacity.tw.	77,478
22	incorporat*.tw.	78,384
23	adher*.tw.	34,556
24	((polic* or practice* or program* or innovation*) adj5 (performance or feedback or prompt* or reminder* or incentive* or penalt* or communicat* or social market* or professional development or network* or leadership or opinion leader* or consensus process* or change manage* or train* or audit*)).tw.	87,456
25	integrat*.tw.	216,469
26	scal* up.tw.	1,994
27	Quality Control/	1,438
28	quality of services/	6,031
29	program evaluation/	12,201
30	5 or 6 or 7 or 8 or 9 or 10 or 11 or 12 or 13 or 14 or 15 or 16 or 17 or 18 or 19 or 20 or 21 or 22 or 23 or 24 or 25 or 26 or 27 or 28 or 29	1,137,067
31	exp Obesity/	22,903
32	Weight Gain/	2,925
33	exp Weight Loss/	3,426
34	obes*.tw.	38,244
35	(weight gain or weight loss).tw.	19,875
36	(overweight or over weight or overeat* or over eat*).tw.	16,413
37	weight change*.tw.	2,122
38	((bmi or body mass index) adj2 (gain or loss or change)).tw.	764
39	(primary prevention or secondary prevention).tw.	5,844
40	(preventive measure* or preventative measure*).tw.	2,815
41	(preventive care or preventative care).tw.	1,198
42	(obes* adj2 (prevent* or treat*)).tw.	4,884
43	31 or 32 or 33 or 34 or 35 or 36 or 37 or 38 or 39 or 40 or 41 or 42	65,396
44	exp Exercise/	24,563
45	physical activity.tw.	31,222
46	physical inactivity.tw.	1,843
47	(“physical education” or “physical training”).tw.	6,220
48	Physical Fitness/	4,077
49	sedentary.tw.	6,296
50	exp Sports/	24,756
51	Dance/	2,120
52	(dance* or dancing).tw.	7,867
53	(exercise* adj2 aerobic*).tw.	2,033
54	sport*.tw.	33,287
55	((lifestyle* or life style*) adj5 activ*).tw.	2,255
56	44 or 45 or 46 or 47 or 48 or 49 or 50 or 51 or 52 or 53 or 54 or 55	98,946
57	nutrition*.tw.	24,725
58	(health* adj2 eat*).tw.	3,701
59	fruit*.tw.	17,266
60	vegetable*.tw.	5,508
61	canteen*.tw.	132
62	food service*.tw.	521
63	(diet* or food habits or fat or menu*).tw.	51,336
64	(calorie or calories or kilojoule*).tw.	3,936
65	Food Intake/	14,041
66	energy density.tw.	300
67	Eating/	11,764
68	Feeding Behavior/ or feeding behavio?r*.mp.	11,191
69	dietary intake.tw.	2,104
70	Food/	13,443
71	Carbonated Beverages/ or soft drink*.mp.	684
72	soda.tw.	418
73	sweetened drink*.tw.	52
74	confectionar*.tw.	40
75	(school adj (lunch* or meal*)).tw.	538
76	((feeding or food or nutrition*) adj program*).tw.	988
77	cafeteria*.tw.	720
78	57 or 58 or 59 or 60 or 61 or 62 or 63 or 64 or 65 or 66 or 67 or 68 or 69 or 70 or 71 or 72 or 73 or 74 or 75 or 76 or 77	114,688
79	smok*.tw.	52,934
80	Nicotine/	10,600
81	Tobacco smoking/	29,571
82	((ceas* or cess* or prevent* or stop* or quit* or abstin* or abstain* or reduc*) adj5 (smok* or tobacco or nicotine)).tw.	22,093
83	“Tobacco Use Disorder”/	198
84	ex-smoker*.tw.	712
85	anti-smok*.tw.	540
86	79 or 80 or 81 or 82 or 83 or 84 or 85	60,113
87	alcohol drinking/ or binge drinking/	2,226
88	alcohol*.tw.	125,592
89	Alcoholic Intoxication/ or Alcoholism/	29,153
90	drink*.tw.	50,966
91	liquor*.tw.	890
92	beer*.tw.	2,708
93	wine*.tw.	2,634
94	spirit*.tw.	47,011
95	drunk*.tw.	3,653
96	intoxicat*.tw.	9,133
97	binge.tw.	11,559
98	87 or 88 or 89 or 90 or 91 or 92 or 93 or 94 or 95 or 96 or 97	200,599
99	43 or 56 or 78 or 86 or 98	458,108
100	clinical trial/ or controlled clinical trial/	11,288
101	placebo/	5,228
102	Research Design/	10,996
103	Intervention/	58,664
104	exp Longitudinal Studies/	16,128
105	((Cross-Over or evaluation or comparative) adj Stud*).tw.	17,808
106	clinical trial.tw.	13,637
107	latin square.tw.	493
108	(time adj series).tw.	7,680
109	(before adj2 after adj3 (stud* or trial* or design*)).tw.	2,286
110	((singl* or doubl* or trebl* or tripl*) adj5 (blind* or mark)).tw.	24,988
111	placebo*.tw.	38,937
112	random*.tw.	187,356
113	(matched adj (communit* or school* or population*)).tw.	424
114	control*.tw.	658,383
115	comparison group*.tw.	12,778
116	matched pairs.tw.	1,272
117	outcome stud*.tw.	4,759
118	(qua?iexperimental or qua?i experimental or pseudo experimental).tw.	10,839
119	(nonrandomi?ed or non randomi?ed or psuedo randomi?ed or quasi randomi?ed).tw.	2,226
120	prospectiv*.tw.	64,156
121	volunteer*.tw.	37,238
122	100 or 101 or 102 or 103 or 104 or 105 or 106 or 107 or 108 or 109 or 110 or 111 or 112 or 113 or 114 or 115 or 116 or 117 or 118 or 119 or 120 or 121	945,144
123	(child or children or adolescen* or teen*).tw.	754,758
124	4 and 30 and 99 and 122 and 123	1,049
125	limit 124 to up=20160901-20190412	184

## CUMULATIVE INDEX TO NURSING AND ALLIED HEALTH LITERATURE

**Table:**

#	Query	Results
S1	(MH “Schools”) OR (MH “Schools, Elementary”) OR (MH “Schools, Middle”) OR (MH “Schools, Secondary”)	20,192
S2	((primary or elementary or middle or junior or high or secondary) n1 (school* or student*))	42,756
S3	kinder*	3,121
S4	S1 OR S2 OR S3	54,176
S5	implement*	163,174
S6	dissemin*	19,963
S7	adopt*	54,029
S8	((polic* or practice* or program* or innovation*) n5 (performance or feedback or prompt* or reminder* or incentive* or penalt* or communicat* or “social market*” or “professional development” or network* or leadership or “opinion leader*” or “consensus process*” or “change manage*” or train* or audit*))	60,829
S9	“organi?ational change*”	12,260
S10	diffus*	40,947
S11	“system* change*”	2,038
S12	“quality improvement*”	52,948
S13	transform*	37,570
S14	translat*	47,031
S15	transfer*	68,493
S16	uptake*	33,290
S17	sustainab*	15,336
S18	institutionali*	7,551
S19	routin*	77,827
S20	maintenance	45,478
S21	capacity	61,690
S22	incorporat*	52,472
S23	adher*	55,246
S24	practice	565,324
S25	integrat*	108,963
S26	“scal* up”	3,043
S27	S5 OR S6 OR S7 OR S8 OR S9 OR S10 OR S11 OR S12 OR S13 OR S14 OR S15 OR S16 OR S17 OR S18 OR S19 OR S20 OR S21 OR S22 OR S23 OR S24 OR S25 OR S26	1,209,243
S28	(MH “Obesity+”)	84,796
S29	(MH “Weight Gain”)	10,596
S30	(MH “Weight Loss”)	18,829
S31	obes*	113,508
S32	(“weight gain” or “weight loss”)	45,507
S33	(overweight or “over weight” or overeat* or “over eat*”)	26,427
S34	“weight change*”	3,356
S35	((bmi or body mass index) n2 (gain or loss or change))	2,901
S36	“Primary Prevention”	5,678
S37	“secondary prevention”	5,003
S38	“preventive measure*”	4,270
S39	“preventative measure*”	753
S40	“preventive care” or “preventative care”	2,585
S41	(obes* n2 (prevent* or treat*))	18,976
S42	S28 OR S29 OR S30 OR S31 OR S32 OR S33 OR S34 OR S35 OR S36 OR S37 OR S38 OR S39 OR S40 OR S41	162,511
S43	(MH “Exercise+”)	98,166
S44	(MH “Physical Activity”)	34,052
S45	“physical inactivity”	2,862
S46	(MH “Motor Activity+”)	11,331
S47	(MH “Physical Education and Training”) OR “physical education” or “physical training”	5,789
S48	(MH “Physical Fitness”)	15,398
S49	“sedentary”	12,921
S50	(MH “Life Style+”) OR (MH “Life Style, Sedentary”)	191,970
S51	(MH “Leisure Activities+”)	59,776
S52	(MH “Sports+”)	70,276
S53	(MH “Dancing+”) OR “Dance*”	5,011
S54	(exercise* n1 aerobic*)	8,133
S55	sport*	60,320
S56	((lifestyle* or “life style*”) n5 activ*)	3,102
S57	S43 OR S44 OR S45 OR S46 OR S47 OR S48 OR S49 OR S50 OR S51 OR S52 OR S53 OR S54 OR S55 OR S56	422,446
S58	(MH “Diet+”)	99,721
S59	nutrition*	132,059
S60	health* n2 eat*	6,335
S61	“Child Nutrition Sciences” OR (MH “Child Nutrition”)	6,615
S62	(MH “Fruit+”)	22,055
S63	(MH “Vegetables”) OR “vegetable*”	19,045
S64	fruit*	23,015
S65	canteen*	222
S66	(MH “Food Services”) OR “food service*”	7,038
S67	“menu*”	3,133
S68	“calorie” or calories or kilojoule*	6,393
S69	(MH “Energy Intake”)	15,666
S70	(MH “Energy Density”)	768
S71	(MH “Eating”)	5,602
S72	(MH “Eating Behavior”) OR “feeding behavio?r*”	13,107
S73	(MH “Food Intake”) OR “dietary intake”	15,815
S74	(MH “Food Habits”)	11,568
S75	(MH “Food”)	12,475
S76	(MH “Carbonated Beverages”) OR “soft drink*”	2,878
S77	“soda”	932
S78	“sweetened drink*”	157
S79	(MH “Dietary Fats”)	11,637
S80	“confectionar*”	64
S81	(MH “Candy”)	550
S82	(school n1 (lunch* or meal*))	997
S83	((feeding or food or nutrition*) n1 program*)	3,598
S84	“cafeteria*”	553
S85	(MH “Nutritional Status”)	12,624
S86	S58 OR S59 OR S60 OR S61 OR S62 OR S63 OR S64 OR S65 OR S66 OR S67 OR S68 OR S69 OR S70 OR S71 OR S72 OR S73 OR S74 OR S75 OR S76 OR S77 OR S78 OR S79 OR S80 OR S81 OR S82 OR S83 OR S84 OR S85	254,598
S87	(MH “Smoking+”)	61,753
S88	(MH “Smoking Cessation Programs”) OR (MH “Tobacco Abuse Control (Saba CCC)”) OR (MH “Tobacco Abuse (Saba CCC)”) OR “Tobacco Use Cessation”	2,476
S89	smok*	102,107
S90	(MH “Nicotine”)	3,843
S91	(MH “Tobacco”)	6,806
S92	((ceas* or cess* or prevent* or stop* or quit* or abstin* or abstain* or reduc*) n5 (smok* or tobacco or nicotine))	34,452
S93	(MH “Substance Use Disorders”)	30,133
S94	“ex-smoker*”	865
S95	“anti-smok*”	507
S96	S87 OR S88 OR S89 OR S90 OR S91 OR S92 OR S93 OR S94 OR S95	131,288
S97	(MH “Binge Drinking”) OR (MH “Drinking Behavior”)	2,983
S98	alcohol*	88,324
S99	(MH “Alcoholism”) OR (MH “Alcoholic Intoxication”)	17,169
S100	drink*	49,157
S101	liquor*	648
S102	beer*	1,813
S103	wine*	2,861
S104	spirit*	26,997
S105	drunk*	1,346
S106	intoxicat*	6,974
S107	binge	6,400
S108	S97 OR S98 OR S99 OR S100 OR S101 OR S102 OR S103 OR S104 OR S105 OR S106 OR S107	136,966
S109	S42 OR S57 OR S86 OR S96 OR S108	917,121
S110	(MH “Randomized Controlled Trials”) OR (MH “Clinical Trials+”)	257,461
S111	(MH “Random Assignment”)	54,198
S112	(MH “Double-Blind Studies”)	40,941
S113	(MH “Single-Blind Studies”)	12,411
S114	(MH “Placebos”)	11,197
S115	(MH “Study Design”)	28,853
S116	(MH “Experimental Studies”) OR “Intervention Studies”	26,918
S117	(MH “Evaluation Research”) OR “Evaluation Studies”	112,449
S118	(MH “Comparative Studies”)	186,640
S119	(MH “Prospective Studies”) OR “Longitudinal Studies”	383,320
S120	(MH “Crossover Design”) OR “Cross-Over Studies”	17,483
S121	“clinical trial*”	207,810
S122	“latin square”	191
S123	(MH “Time Series”)	2,468
S124	(before n2 after n3 (stud* or trial* or design*))	5,063
S125	((singl* or doubl* or trebl* or tripl*) n5 (blind* or mark))	65,046
S126	placebo*	54,518
S127	random*	343,356
S128	(matched n1 (communit* or school* or population*))	1,484
S129	control*	1,068,659
S130	“comparison group*”	6,675
S131	“matched pairs”	1,481
S132	“outcome stud*”	2,824
S133	qua?iexperimental or “qua?i experimental” or “pseudo experimental”	13,804
S134	nonrandomi?ed or “non randomi?ed” or “psuedo randomi?ed” or “qua?i randomi?ed”	8,381
S135	prospectiv*	449,494
S136	volunteer*	44,312
S137	S110 OR S111 OR S112 OR S113 OR S114 OR S115 OR S116 OR S117 OR S118 OR S119 OR S120 OR S121 OR S122 OR S123 OR S124 OR S125 OR S126 OR S127 OR S128 OR S129 OR S130 OR S131 OR S132 OR S133 OR S134 OR S135 OR S136	1,733,188
S138	(MH “Child”) OR (MH “Adolescence”)	667,718
S139	(child or children or adolescen* or teen*)	876,565
S140	S138 OR S139	876,565
S141	S4 AND S27 AND S109 AND S137 AND S140 (limited to September 2016-April 2019)	656

## EDUCATION RESOURCE INFORMATION CENTER

noft((School* **OR** ((primary **OR** elementary **OR** middle **OR** junior **OR** high **OR** secondary) **AND** student*) **OR** kinder*)) **AND** noft((Implement* **OR** dissemin* **OR** adopt* **OR** practice* **OR** “organisational change*” **OR** “organizational change*” **OR** diffuse* **OR** “system* change*” **OR** “quality improvement*” **OR** transform* **OR** translat* **OR** transfer* **OR** uptake* **OR** sustainab* **OR** institutionali* **OR** routin* **OR** maintenance **OR** capacity **OR** incorporate* **OR** adher* **OR** program* **OR** integrat* **OR** “scal* up”)) **AND** noft((Obes* **OR** “Weight Gain” **OR** “Weight Loss” **OR** overweight **OR** “over weight” **OR** overeat* **OR** “over eat*” **OR** “weight change*” **OR** ((bmi **OR** body mass index) **AND** (gain **OR** loss **OR** change)) **OR** “Primary Prevention” **OR** “secondary prevention” **OR** “preventive measure*” **OR** “preventative measure” **OR** “preventive care” **OR** “preventative care” **OR** Exercise **OR** “physical activity” **OR** “physical inactivity” **OR** “Motor Activity” **OR** “physical education” or “physical training” **OR** “Physical Fitness” **OR** sedentary **OR** “Life Style” **OR** lifestyle **OR** “Leisure Activit*” **OR** sport* **OR** Dancing **OR** dance* **OR** aerobic* **OR** diet **OR** nutrition* **OR** “Child Nutrition Sciences” **OR** fruit* **OR** vegetable* **OR** canteen* **OR** “food service*” **OR** menu* **OR** calorie* **OR** kilojoule* **OR** “Energy Intake” **OR** “energy density” **OR** eating **OR** “Feeding Behavio*” **OR** “dietary intake” **OR** food **OR** “Carbonated Beverage*” **OR** “soft drink*” **OR** soda **OR** “sweetened drink*” **OR** “Dietary Fats” **OR** confectionar* **OR** “school lunch*” **OR** “school meal*” **OR** ((feeding **OR** food **OR** nutrition*) **AND** program*) **OR** cafeteria* **OR** smok* **OR** Tobacco **OR** Nicotine **OR** alcohol* **OR** drink* **OR** liquor* **OR** beer* **OR** wine* **OR** spirit* **OR** drunk* **OR** intoxicat* **OR** binge)) **AND** noft((“clinical trial*” **OR** random* **OR** placebo* **OR** “Research Design” **OR** “Intervention Stud*” **OR** “Evaluation Stud*” **OR** “Comparative Stud*” **OR** “Longitudinal Stud*” **OR** “Cross-Over Stud*” **OR** “latin square” **OR** “time series” **OR** ((before **AND** after) **AND** (stud* **OR** trial* **OR** design*)) **OR** ((singl* **OR** doubl* **OR** trebl* **OR** tripl*) **AND** (blind* **OR** mark)) **OR** (matched **AND** (communit* **OR** school* **OR** population*)) **OR** control* **OR** “comparison group*” **OR** “control group*” **OR** “matched pairs” **OR** “outcome stud*” **OR** qu?siexperimental **OR** “qua?i experimental” **OR** “pseudo experimental” **OR** nonrandomized **OR** nonrandomised **OR** prospective* **OR** volunteer*)) **AND** noft(Child or children or teen* or adolescen*)

## DISSERTATIONS AND THESES

Ab/ti/su((School* **OR** ((primary **OR** elementary **OR** middle **OR** junior **OR** high **OR** secondary) **AND** student*) **OR** kinder*)) **AND** Ab/ti/su ((Implement* **OR** dissemin* **OR** adopt* **OR** practice* **OR** “organisational change*” **OR** “organizational change*” **OR** diffuse* **OR** “system* change*” **OR** “quality improvement*” **OR** transform* **OR** translat* **OR** transfer* **OR** uptake* **OR** sustainab* **OR** institutionali* **OR** routin* **OR** maintenance **OR** capacity **OR** incorporate* **OR** adher* **OR** program* **OR** integrat* **OR** “scal* up”)) **AND** Ab/ti/su ((Obes* **OR** “Weight Gain” **OR** “Weight Loss” **OR** overweight **OR** “over weight” **OR** overeat* **OR** “over eat*” **OR** “weight change*” **OR** ((bmi **OR** body mass index) **AND** (gain **OR** loss **OR** change)) **OR** “Primary Prevention” **OR** “secondary prevention” **OR** “preventive measure*” **OR** “preventative measure” **OR** “preventive care” **OR** “preventative care” **OR** Exercise **OR** “physical activity” **OR** “physical inactivity” **OR** “Motor Activity” **OR** “physical education” **OR** “physical training” **OR** “Physical Fitness” **OR** sedentary **OR** “Life Style” **OR** lifestyle **OR** “Leisure Activit*” **OR** sport* **OR** Dancing **OR** dance* **OR** aerobic* **OR** diet **OR** nutrition* **OR** “Child Nutrition Sciences” **OR** fruit* **OR** vegetable* **OR** canteen* **OR** “food service*” **OR** menu* **OR** calorie* **OR** kilojoule* **OR** “Energy Intake” **OR** “energy density” **OR** eating **OR** “Feeding Behavio*” **OR** “dietary intake” **OR** food **OR** “Carbonated Beverage*” **OR** “soft drink*” **OR** soda **OR** “sweetened drink*” **OR** “Dietary Fats” **OR** confectionar* **OR** “school lunch*” **OR** “school meal*” **OR** ((feeding **OR** food **OR** nutrition*) **AND** program*) **OR** cafeteria* **OR** smok* **OR** Tobacco **OR** Nicotine **OR** alcohol* **OR** drink* **OR** liquor* **OR** beer* **OR** wine* **OR** spirit* **OR** drunk* **OR** intoxicat* **OR** binge)) **AND** Ab/ti/su ((“clinical trial*” **OR** random* **OR** placebo* **OR** “Research Design” **OR** “Intervention Stud*” **OR** “Evaluation Stud*” **OR** “Comparative Stud*” **OR** “Longitudinal Stud*” **OR** “Cross-Over Stud*” **OR** “latin square” **OR** “time series” **OR** ((before **AND** after) **AND** (stud* **OR** trial* **OR** design*)) **OR** ((singl* **OR** doubl* **OR** trebl* **OR** tripl*) **AND** (blind* **OR** mark)) **OR** (matched **AND** (communit* **OR** school* **OR** population*)) **OR** control* **OR** “comparison group*” **OR** “control group*” **OR** “matched pairs” **OR** “outcome stud*” **OR** qu?siexperimental **OR** “qua?i experimental” **OR** “pseudo experimental” **OR** nonrandomized **OR** nonrandomised **OR** prospective* **OR** volunteer*)) **AND** Ab/ti/su ((Child **OR** children **OR** teen* **OR** adolescen*))

## SCOPUS

**TITLE** (school* **OR** ((primary **OR** elementary **OR** middle **OR** junior **OR** high **OR** secondary) **AND** student*) **OR** kinder*) **AND ABS** (implement* **OR** dissemin* **OR** adopt* **OR** practice* **OR** “organisational change*” **OR** “organizational change*” **OR** diffuse* **OR** “system* change*” **OR** “quality improvement*” **OR** transform* **OR** translat* **OR** transfer* **OR** uptake* **OR** sustainab* **OR** institutionali* **OR** routin* **OR** maintenance **OR** capacity **OR** incorporate* **OR** adher* **OR** program* **OR** integrat* **OR** “scal* up”) **AND TITLE** (obes* **OR** “Weight Gain” **OR** “Weight Loss” **OR** overweight **OR** “over weight” **OR** overeat* **OR** “over eat*” **OR** “weight change*” **OR** ((bmi **OR** body **AND** mass **AND** index) **AND** (gain **OR** loss **OR** change)) **OR** “Primary Prevention” **OR** “secondary prevention” **OR** “preventive measure*” **OR** “preventative measure” **OR** “preventive care” **OR** “preventative care” **OR** exercise **OR** “physical activity” **OR** “physical inactivity” **OR** “Motor Activity” **OR** “physical education” **OR** “physical training” **OR** “Physical Fitness” **OR** sedentary **OR** “Life Style” **OR** lifestyle **OR** “Leisure Activit*” **OR** sport* **OR** dancing **OR** dance* **OR** aerobic* **OR** diet **OR** nutrition* **OR** “Child Nutrition Sciences” **OR** fruit* **OR** vegetable* **OR** canteen* **OR** “food service*” **OR** menu* **OR** calorie* **OR** kilojoule* **OR** “Energy Intake” **OR** “energy density” **OR** eating **OR** “Feeding Behavio*” **OR** “dietary intake” **OR** food **OR** “Carbonated Beverage*” **OR** “soft drink*” **OR** soda **OR** “sweetened drink*” **OR** “Dietary Fats” **OR** confectionar* **OR** “school lunch*” **OR** “school meal*” **OR** ((feeding **OR** food **OR** nutrition*) **AND** program*) **OR** cafeteria* **OR** smok* **OR** tobacco **OR** nicotine **OR** alcohol* **OR** drink* **OR** liquor* **OR** beer* **OR** wine* **OR** spirit* **OR** drunk* **OR** intoxicat* **OR** binge) **AND ABS** ((“clinical trial*” **OR** random* **OR** placebo* **OR** “Research Design” **OR** “Intervention Stud*” **OR** “Evaluation Stud*” **OR** “Comparative Stud*” **OR** “Longitudinal Stud*” **OR** “Cross-Over Stud*” **OR** “latin square” **OR** “time series” **OR** ((before **AND** after) **AND** (stud* **OR** trial* **OR** design*)) **OR** ((singl* **OR** doubl* **OR** trebl* **OR** tripl*) **AND** (blind* **OR** mark)) **OR** (matched **AND** (communit* **OR** school* **OR** population*)) **OR** control* **OR** “comparison group*” **OR** “control group*” **OR** “matched pairs” **OR** “outcome stud*” **OR** qu?siexperimental **OR** “qua?i experimental” **OR** “pseudo experimental” **OR** nonrandomized **OR** nonrandomised **OR** prospective* **OR** volunteer*)) **AND** TITLE (child **OR** children **OR** teen* **OR** adolescen*) **AND** (LIMIT-TO (PUBYEAR, 2019) **OR LIMIT-TO** (**PUBYEAR**, 2018) **OR** LIMIT-TO (PUBYEAR, 2017) **OR** LIMIT-TO (**PUBYEAR**, 2016))

## COCHRANE LIBRARY

(School* **OR** kinder* **OR** ((primary **OR** elementary **OR** middle **OR** junior **OR** high **OR** secondary) **AND** student*)) in Title Abstract Keyword **AND** (Implement* **OR** dissemin* **OR** adopt* **OR** practice* **OR** “organisational change*” **OR** “organizational change*” **OR** diffuse* **OR** “system* change*” **OR** “quality improvement*” **OR** transform* **OR** translat* **OR** transfer* **OR** uptake* **OR** sustainab* **OR** institutionali* **OR** routin* **OR** maintenance **OR** capacity **OR** incorporate* **OR** adher* **OR** program* **OR** integrat* **OR** “scal* up”) in Title Abstract Keyword **AND** (Obes* **OR** “Weight Gain” **OR** “Weight Loss” **OR** overweight **OR** “over weight” **OR** overeat* **OR** “over eat*” **OR** “weight change*” **OR** ((bmi **OR** body mass index) **AND** (gain **OR** loss **OR** change)) **OR** “Primary Prevention” **OR** “secondary prevention” **OR** “preventive measure*” **OR** “preventative measure” **OR** “preventive care” **OR** “preventative care” **OR** Exercise **OR** “physical activity” **OR** “physical inactivity” **OR** “Motor Activity” **OR** “physical education” or “physical training” **OR** “Physical Fitness” **OR** sedentary **OR** “Life Style” **OR** lifestyle **OR** “Leisure Activit*” **OR** sport* **OR** Dancing **OR** dance* **OR** aerobic* **OR** diet **OR** nutrition* **OR** “Child Nutrition Sciences” **OR** fruit* **OR** vegetable* **OR** canteen* **OR** “food service*” **OR** menu* **OR** calorie* **OR** kilojoule* **OR** “Energy Intake” **OR** “energy density” **OR** eating **OR** “Feeding Behavio*” **OR** “dietary intake” **OR** food **OR** “Carbonated Beverage*” **OR** “soft drink*” **OR** soda **OR** “sweetened drink*” **OR** “Dietary Fats” **OR** confectionar* **OR** “school lunch*” **OR** “school meal*” **OR** ((feeding **OR** food **OR** nutrition*) **AND** program*) **OR** cafeteria* **OR** smok* **OR** Tobacco **OR** Nicotine **OR** alcohol* **OR** drink* **OR** liquor* **OR** beer* **OR** wine* **OR** spirit* **OR** drunk* **OR** intoxicat* **OR** binge) in Title Abstract Keyword **AND** Child or children or teen* or adolescen* in Title Abstract Keyword - with Cochrane Library publication date Between Sep 2016 and Apr 2019, in Cochrane Reviews, Cochrane Protocols, Trials (Word variations have been searched)

## Appendix A3

**Table:** Implementation strategies as characterized by effective practice and organization of care (EPOC) taxonomy list

Category	Subcategory	Definition
Interventions targeted at health care organizations	Organizational culture	Strategies to change organizational culture
Interventions targeted at health care workers	Audit and feedback	A summary of health workers’ performance over a specified period of time, given to them in a written electronic or verbal format. The summary may include recommendations for clinical action.
	Clinical incident reporting	System for reporting critical incidents
	Monitoring the performance of the delivery of healthcare	Monitoring of health services by individuals or health care organizations, for example, by comparing with an external standard
	Communities of practice	Groups of people with a common interest who deepen their knowledge and expertise in this area by interacting on an ongoing basis
	Continuous quality improvement	An iterative process to review and improve care that includes involvement of health care teams, analysis of a process or system, a structured process improvement method or problem solving approach, and use of data analysis to assess changes
	Educational games	The use of games as an educational strategy to improve standards of care
	Educational materials	Distribution to individuals, or groups, of educational materials to support clinical care, that is, any intervention in which knowledge is distributed. For example, this may be facilitated by the internet, learning critical appraisal skills; skills for electronic retrieval of information, diagnostic formulation; question formulation
	Educational meetings	Courses, workshops, conferences, or other educational meetings
	Educational outreach visits, or academic detailing	Personal visits by a trained person to health workers in their own settings to provide information with the aim of changing practice.
	Clinical practice guidelines	Clinical guidelines are systematically developed statements to assist health care providers and patients to decide on appropriate health care for specific clinical circumstances (U.S. IOM). Within schools, this may include the development of best-practice nutrition or physical activity guidelines, which are then provided to school staff as instructional material to support the implementation of policies, practices, or programs.
	Interprofessional education	Continuing education for health professionals that involves more than one profession in joint, interactive learning
	Local consensus processes	Formal or informal local consensus processes, for example, agreeing a clinical protocol to manage a patient group, adapting a guideline for a local health system or promoting the implementation of guidelines
	Local opinion leaders	The identification and use of identifiable local opinion leaders to promote good clinical practice
	Managerial supervision	Routine supervision visits by health staff
	Patient-mediated interventions	The use of patients, for example, by providing patient outcomes, to change professional practice
	Public release of performance data	Informing the public about health care providers by the release of performance data in written or electronic form
	Reminders	Manual or computerized interventions that prompt health workers to perform an action during a consultation with a patient, for example, computer decision support systems
	Routine patient-reported outcome measures	Routine administration and reporting of patient-reported outcome measures to providers and/or patients
	Tailored interventions	Interventions to change practice that are selected based on an assessment of barriers to change, for example, through interviews or surveys

## Appendix A4

**Table:** Data extraction table for included studies

Alaimo et al. [43]		
Study characteristics	Design: nonrandomized trial	
	Setting: Middle school	
	Population: Michigan, USA	
*n* (number of participants)	Baseline: HSAT: 24 (schools); HSAT + SNAK: 5 (schools); HSAT + MSBE: 25 (schools);Control: 21 (schools)	Follow-up: HSAT: 18 (schools); HSAT + SNAK: 5 (schools); HSAT + MSBE: 22 (schools);Control: 20 (schools)
Intervention description (*implementation strategy* categorized by EPOC)	Intervention description: Healthy School Actions Tools (HSAT) included questions on the following topics: school nutrition policies, school nutrition environment, school health education programs including nutrition education, and food service programs. - At the end of each module, schools were to brainstorm several “bright ideas” they could implement. Schools identified bright ideas for each section were shown together to facilitate the development of an action plan with goals (*tailored interventions*). - Schools were asked to prioritize goals and received funding to implement nutrition education or nutrition activities in their action plans and compensate for any loss in canteen revenue (*external funding*). - Schools were provided with a facilitator meeting to assess nutrition environments and policies (*educational outreach visits*). - Guidance documents were provided to school staff (*educational materials*). - Schools established a coordinated school health team (local consensus approach). - Implementation of policy in cafeteria à la carte lines (*clinical practice guidelines*).	
Comparator description	Usual practice or waitlist control	
Implementation outcomes	Outcome: mean nutrition policy score (range 0–6) and mean nutrition education and/or practice change score (range 0–14)	
	Measure: The Middle-School School Environment and Policy Survey was completed either online or by paper. There were two versions of the survey: one for administrators and one for food service directors.	
	Results: Median (range) nutrition policy score: 0.65 (0.2–1.1)	
Cunningham-Sabo et al. [44]		
Study characteristics	Design: RCT	
	Setting: Elementary school	
	Population: New Mexico and South Dakota, USA	
*n* (number of participants)	Baseline: Intervention: 19 (schools); Control: 20 (schools)	Follow-up: Intervention: 19 (schools); Control: 20 (schools)
Intervention description (*implementation strategy* categorized by EPOC)	Intervention description: Components included a classroom curriculum, new skills and activities for the school physical education classes, activities and events with the families, and skill building with school food service - The intervention included the development of nutrient guidelines operationalized as behavioral guidelines (*clinical practice guidelines*). Training sessions were conducted twice each school year with food service staff. Schools also received at least five kitchen visits in the first year and eight or more visits to each school in the second and third years (*educational outreach visits*). - Materials and activities for the training sessions and kitchen visits were developed (educational materials). - The food service working group met annually and held monthly conference calls to establish and carry out the intervention (educational meetings).	
Comparator description	Usual practice	
Implementation outcomes	Outcome: percentage calories total fat breakfast (%) and percentage energy from total fat Lunch (%)	
	Measure: All schools had a Pathways notebook with forms to be completed for each meal per day. On the form each food item was listed with a complete description of the food, the serving size, and the number of students served the food. Separate forms were completed for breakfast and lunch.	
	Results: Median (range) nutrient content of school meals % of calories from fat breakfast/ lunch: −3% (−3.3% to −2.7%)	
Delk et al. [46]		
Study characteristics	Design: Cluster-RCT	
	Setting: Middle schools	
	Population: Central Texas, USA	
*n* (number of participants)	Baseline: Basic: 10 (schools); Basic Plus: 10 (schools); Basic Plus SM: 10 (schools)	Follow-up: 10 (schools); Basic Plus: 10 (schools); Basic Plus SM: 10 (schools)
Intervention description (*implementation strategy* categorized by EPOC)	Intervention description: Promote the adoption of active breaks (ABs) by teachers. - In promoting ABs, we developed a CATCH Middle School ABs guide. Each school received 10 hard copies of the ABs and an electronic version. ABs stated the amount of time required, contained instructional content, identified equipment needed (clinical practice guidelines). - A CATCH Team was developed at each school, consisting of staff members, parents and community members (local consensus approach) - Schools were required to send representatives from their CATCH Team to 8 trainings and were assigned a CATCH facilitator to conduct visits at these schools (educational meetings). - A CATCH facilitator was assigned and conducted monthly visits at these schools. During these visits strategies to help CATCH Teams promote ABs on their campus were developed (educational outreach visits and tailored interventions). - Social marketing campaigns to promote physical activity (other)	
Comparator description	Different implementation strategy	
Implementation outcomes	Outcome: Teacher reported frequency of AB implementation including: Have you conducted at least one AB this year; Percentage of teachers that conducted activity breaks weekly (%, N); Last week, did you conduct an activity break on at least 1 day? (%, N)	
	Measure: The survey is a 15-item, self-administered questionnaire that includes items on teacher implementation of ABs, encouragement of specific health behaviors, and other process evaluation measures for the CATCH program	
	Results: Median (range) % of teachers that conducted activity breaks weekly: 13.3% (11.1% to 15.4%); Median (range) % implementing a variety of policies and practices: 26.5% (19.4% to 31.9%)	
French et al. [47]		
Study characteristics	Design: Cluster-RCT	
	Setting: High school	
	Population: Minneapolis, USA	
*n* (number of participants)	Baseline: Intervention: 10 (schools); Control: 10 (schools)	Follow-up: Intervention: 10 (schools); Control: 10 (schools)
Intervention description (*implementation strategy* categorized by EPOC)	Intervention description: Increasing the availability of lower-fat foods in cafeteria à la carte areas and implementing school-wide, student-based promotions of these lower-fat foods. The goal was to increase lower-fat à la carte food availability by 30% relative to baseline. The ultimate goal was to have 50% of products be lower fat. - Quarterly meetings between researchers and staff were held to review progress toward goals (*local consensus approach*). - Development of tailored lists of high and low fat foods for schools (*tailored interventions*) - Staff worked with the student groups and their faculty advisors to train the students for specific promotional activities (*educational meetings*) - Student groups were offered financial incentives for completing each promotion (*pay for performance*)	
Comparator description	Usual practice or waitlist control	
Implementation outcomes	Outcome: Students seen any posters in school about cafeteria food choices; Students heard any messages over public address system, in school; Students heard about any contests or events at school about cafeteria food choices; Students took part in any taste tests, food samplings, or contests in the school cafeteria; Percentage low-fat à la carte foods	
	Measure: Complete à la carte inventories in intervention and control schools were conducted by trained research staff at baseline and after the second intervention year	
	Results: Median (range) % of program implementation: 33% (11% to 41%)	
Heath and Coleman [48]		
Study characteristics	Design: nonrandomized trial	
	Setting: Elementary schools	
	Population: West Texas and East New Mexico, USA	
*n* (number of participants)	Baseline: Intervention: 20 (schools); Control: 4 (schools)	Follow-up: Intervention: 20 (schools); Control: 4 (schools)
Intervention description (*implementation strategy* categorized by EPOC)	Intervention description: CATCH intervention was delivered at school level to: reduce the total fat content of food served to 30% and reduce the total sodium content to 600 mg ‐ 1000 mg per serving. - Staff received training sessions (*educational meetings*). - Staff received ongoing support visits to implement EATSMART/ CATCH PE (*educational outreach visits*). Educational materials were provided to staff/schools. Smart choices manual was provided to all schools (*educational materials*).	
Comparator description	Usual practice	
Implementation outcomes	Outcome: % fat in breakfast; Sodium (mg) in breakfast; % fat in lunch; Sodium (mg) in lunch	
	Measure: Menus and recipes were collected for 5 consecutive days during each semester in every year of the study. Recipes for these menus were obtained by interviewing cooks and kitchen managers in school cafeterias and by reviewing the cafeteria production sheets for each meal. Foods from the menus, production sheets, and recipes were entered into a nutritional database that is especially useful for ethnic foods. Once the nutrient content of the meals was analyzed, averages of breakfast and lunch values across the 5 days of data collection were obtained.	
	Results: % fat in school meal: −1.7% (−4.4% to 1%); Sodium of school meals: −29.5 (−48 to −11)	
Hoelscher et al. [49]		
Study characteristics	Design: nonrandomized trial	
	Setting: Elementary schools	
	Population: Texas, USA	
*n* (number of participants)	Baseline: Intervention: 15 (schools); Control: 15 (schools)	Follow-up: Intervention: 15 (schools); Control: 15 (schools)
Intervention description (*implementation strategy* categorized by EPOC)	Intervention description: Target multiple aspects of the school environment, including the classroom, nutrition services and the cafeteria environment, physical education activities, family and home environment, and, via school health promotion messages and events, the broader school community. - Coordinated school health CATCH training and booster training sessions and community workshops (educational meetings). - Program materials, CATCH component coordination kit and health promotion resources (educational materials). - CATCH committee meetings (local consensus approach) - Recognition and funds for CATCH through rewards program (pay for performance) - School social marketing efforts (use of information and communication technology) - Family fun night activities (other) - Community member required on CATCH committee and CATCH community workshops (local consensus approach) - CATCH facilitator support visits (2–3 visits/4–6 weeks) (educational outreach visit).	
Comparator description	Different implementation strategy	
Implementation outcomes	Outcome: Continuous: CATCH parent and extracurricular activities, CATCH coordinated healthy eating–related activities, CATCH coordinated physical activity–related activities, Number of CATCH lessons taught, Number health lessons taught Dichotomous: % Reporting CATCH lessons in schoolroom, % Reporting that fruit usually served at lunch	
	Measure: Structured interview with CATCH Champion and self‐administered questionnaire for classroom teachers	
	Results: Mean number of lessons/or activities: 0.8 (−0.4 to 1. 2) % implementing a variety of policies and practices: 4.4% (3.6% to 5.2%)	
Lytle et al. [50]		
Study characteristics	Design: RCT	
	Setting: Middle schools	
	Population: Minneapolis. USA	
*n* (number of participants)	Baseline: 8 (schools); Control: 8 (schools)	Follow-up: 8 (schools); Control: 8 (schools)
Intervention description (*implementation strategy* categorized by EPOC)	Intervention description: Teens Eating for Energy and Nutrition at School (TEENS) was a school‐based intervention trial with a goal of developing and evaluating school and family‐linked intervention strategies to promote students’ consumption of fruit, vegetable, and lower fat snacks. TEENS intervention included classroom, family, school policy, and food service components. - School Nutrition Advisory Councils were established to convene school and parental stakeholders to discuss and propose school-level policy to improve the school food environment (local opinion leaders and local consensus approach). - District food service directors and workers from intervention schools attended training that emphasized the importance of offering more fruit and vegetables, gave them new tools for promoting fruit, vegetable, and lower fat snacks (educational materials and educational meetings).	
Comparator description	Usual practice or waitlist control	
Implementation outcomes	Outcome: Food offered: Foods to limit and foods to promote. Foods sold: Foods to limit and foods to promote	
	Measure: Data collected included the total number of students served the meal pattern lunch, the types and amounts of fruit and vegetable choices offered and sold, and the number of vegetable salads sold. With a few exceptions, these data were extracted from schools’ food production records.	
	Results: % of schools offering or selling targeted foods: 8.5% (4% to 12%)	
Mobley et al. [51]		
Study characteristics	Design: RCT	
	Setting: Middle schools	
	Population: 10 districts across USA	
*n* (number of participants)	Baseline: Intervention: 21 (schools); Control: 21 (schools)	Follow-up: Intervention: 21 (schools); Control: 21 (schools)
Intervention description (*implementation strategy* categorized by EPOC)	Intervention description: Lower the average fat content of food served in schools; serve at least 2 servings of fruit and/or vegetables per student and at least 1 serving per student each day; serve all dessert and snack foods with ≤ 200 kcal per single size serving and/or package; eliminate milk >1% fat, all other added sugar beverages, and 100% fruit juice; serve at least 2 servings of high fiber grain‐based foods and/or legumes per student and at least 1 serving per student each day. - Provision of staff training and educational events (*educational meetings*). - “Taste tests” of new products and unfamiliar foods, including conducting comparison of available items (*educational games*). - Intervention schools received funding to defray expenses and potential loss of income, cafeteria enhancements and to attend training (*external funding*). - Research staff worked with food service managers to identify barriers and develop solutions for schools to achieve selected goals (*tailored interventions*). - Curricula, posters, brief messages displayed near serving lines (*educational materials*). - Research staff met weekly with food service staff to observe the food environment and to plan and support goal achievement (*educational outreach visit*s) - Engagement with social marketing experts to generate content (*use of information and communication in technology*) - Meetings with district level staff and buyers who procure food and with food distributors, to solicit support for change (*other*)	
Comparator description	Usual practice or waitlist control	
Implementation outcomes	Outcome: 12 scores across the following variables: Lower than average fat content; serve two servings of fruit and vegetables; serve all desert and snack foods with <200 kcal; eliminate milk >1% fat; serve at least two servings of high fiber	
	Measure: Data collected by trained staff not involved in the intervention. Nutrition data were extracted from food service management source documents maintained by school food service personnel.	
	Results: % schools meeting various nutrition goals: 15.5% (0% to 88%)	
Nathan et al. [52]		
Study characteristics	Design: nonrandomized trial	
	Setting: Elementary schools	
	Population: NSW, Australia	
*n* (number of participants)	Baseline: Intervention: 407 (schools); Control: 316 (schools)	Follow-up: Intervention: 388 (schools); Control: 258 (schools)
Intervention description (*implementation strategy* categorized by EPOC)	Intervention description: Structured multi-strategy intervention was developed based on theoretical frameworks of practice change and recommendations from reviews and implementation studies conducted in schools and other settings - Program materials, including curriculum resources and information to parents (*educational materials*) - Staff training and professional development (*educational meetings*). - School consensus approach, as well as leadership support and enhancement (*local consensus approach and local opinion leaders*). - School-specific follow-up support (*tailored interventions*) - Incentives in the form of material goods (*other*) - Implementation feedback, including performance monitoring (*monitoring the performance of the delivery of the health care*).	
Comparator description	Minimal support control	
Implementation outcomes	Outcome: The prevalence of vegetable and fruit breaks.	
	Measure: Principal reported computer‐assisted telephone interviewing (CATI).	
	Results: % Schools implementing a vegetable and fruit break: 16.2% (5.6% to 26.8%)	
Nathan et al. [53]		
Study characteristics	Design: RCT	
	Setting: Elementary schools	
	Population: NSW, Australia	
*n* (number of participants)	Baseline: Intervention: 28 (schools); Control: 25 (schools)	Follow-up: Intervention: 27 (schools); Control: 24 (schools)
Intervention description (*implementation strategy* categorized by EPOC)	Intervention description: State government had introduced a healthy school canteen policy. Utilizing a “traffic light” food classification system, the policy classifies foods and beverages sold in school canteens as either “red,” “amber” or “green” based on their nutritional content. For all foods sold in the canteen at recess and lunch the policy requires schools to remove all red foods from regular sale and to fill the menu with green foods and to not let amber foods dominate the menu. - Schools were provided with a written feedback report on their previously supplied canteen menu (*audit and feedback*). - Canteen managers were asked to send an updated version of the menu for review and a second feedback report was generated (*continuous quality improvement*). - Canteen managers were provided with a “Canteen Resource Kit” containing various printed and electronic instructional materials, including electronic menu and pricing templates (*educational materials*) - 1 day (5 hr) group-training workshop was offered to canteen - managers and parent representatives providing education and skill development in the Fresh Tastes@ School policy, label reading, canteen stock and financial management, pricing and promotion, and change management (*educational meeting*) - The workshop provided opportunities for canteen managers to participate in consensus processes through the development of a canteen action plan (*local consensus approach*). - School principals were asked to demonstrate their support for implementation of the Fresh Tastes @ School policy (*local opinion leader*) - The feedback report included a sample “compliant” menu, individually tailored to the schools (*tailored interventions*) - Canteen managers received two support contacts per school term via text messages. These contacts provided targeted advice to overcome common barriers to policy implementation and encouraged canteen managers to review progress against their action plan (*other*).	
Comparator description	Usual practice	
Implementation outcomes	Outcome: Proportion of schools with a menu that did not include red or banned foods and beverages and the proportion of schools where green items make up the majority of the menu (more than 50 % of menu items).	
	Measure: Audits of canteen menus faxed or emailed to the project team by the school.	
	Results: % implementing a variety of policies and practices: 35.5% (30.0% to 41.1%)	
Naylor et al. [54]		
Study characteristics	Design: cluster-RCT	
	Setting: Elementary schools	
	Population: British Columbia, Canada	
*n* (number of participants):	Baseline: Intervention: 7 (schools); Control: 3 (schools)	Follow-up: Intervention: 7 (schools); Control: 3 (schools)
Intervention description (*implementation strategy* categorized by EPOC)	Intervention description: - The AS! BC model provided tools for schools and teachers to create individualized Action Plans that increased PA opportunities across Six Action Zones: School Environment, Scheduled physical education (PE), Classroom Action, Family and Community, Extra‐curricular, Spirit (tailored interventions). - Teachers received teacher-on-call support to attend a Classroom Action training session (half-day) from the AS! BC Support Team and School Facilitators (*educational meetings*) - A committee of school stakeholders, including grade teachers, administrators, parents, health, sport/recreation practitioners, was developed (*local consensus approach*). Teachers had weekly contact with the School Facilitator who would come to the classroom to provide mentorship and demonstrate activities (educational outreach visits).	
Comparator description	Usual practice or waitlist control	
Implementation outcomes	Outcome: Minutes per week of physical activity	
	Measure: Teachers at Intervention schools were asked to complete weekly activity Logs during Phases I and II. Teachers recorded daily, the type, frequency and duration (minutes) of PA implemented in the classroom, in PE or in the other Action Zones. Activity Logs were collected monthly by the School Facilitators.	
	Results: Minutes per week of physical activity implemented in the classroom: 54.9 min (46.4–63.4)	
Perry et al. [56]		
Study characteristics	Design: cluster-RCT	
	Setting: Elementary schools	
	Population: California, Louisiana, Minneapolis and Texas, USA	
*n* (number of participants)	Baseline: CATCH 1: 28 (schools); CATCH 2: 28 (schools); Control: 40 (schools)	Follow-up: CATCH 1: 28 (schools); CATCH 2: 28 (schools); Control: 40 (schools)
Intervention description (*implementation strategy* categorized by EPOC)	Intervention description: EATSMART: to reduce the total fat content of food served to 30%, to reduce the total sodium content to 600‐1000 mg per serving, recommendations to lower the total cholesterol in foods offered. CATCH PE: increase the amount of PE time that students spent in MVPA to 40% of class time. - Staff received training sessions to deliver EATSMART and CATCH PE (*educational meetings*). - Staff received ongoing support visits to implement EATSMART/CATCH PE (*educational outreach visits*). - Educational materials were provided to staff for EATSMART and CATCH PE (*educational materials*). Families were engaged by Family Fun Nights and home curricula (*other*).	
Comparator description	Usual practice or waitlist control	
Implementation outcomes	Outcome: Mean % of kilocalories from fat in lunches, Mean mg of sodium in lunches, cholesterol mg in lunches (mean), quality of PE lesson % of seven activities observed	
	Measure: Quality of PE lesson: Direct observation. Nutrient content: School menu information were collected from schools. Staff conducted in‐person interviews with the cooks about the menus and recipes using standardized probes for ingredients and preparation methods.	
	Results: % of kilocalories from fat in school lunch: −4.3% (−5.8% to −2.8%) Mean mg of sodium in lunches: −100.5 (−167.6 to −33.4) Cholesterol mg in lunches: −8.3 (−16.7 to 0.1) Quality of PE lesson % of seven activities observed: 14.3% (11.6% to 17.0%)	
Perry et al. [55]		
Study characteristics	Design: cluster-RCT	
	Setting: Elementary schools	
	Population: Minnesota, USA	
*n* (number of participants)	Baseline: Intervention: 13 (schools); Control: 13 (schools)	Follow-up: Intervention: 13 (schools); Control: 13 (schools)
Intervention description (*implementation strategy* categorized by EPOC)	Intervention description: Increasing the availability, appeal, and encouragement of fruits and vegetables in the school lunch program; emphasizing changes in the lunch line; and the school snack cart; increase the quality and quantity of fruits and vegetables served; increase the choices of fruits and vegetables in the lunch line, and to vary the type and preparation methods daily; special events to promote fruits and vegetables. - Monthly meetings were held with the cook managers to discuss and share implementation issues and new ideas during the 1st school year (*educational meetings and local consensus approach*). - Intervention staff visited schools weekly, on average, and supported the activities (*educational outreach visits*). - The “High 5 Flyers” that were hung in posters around the school cafeteria (*educational materials*). Sampling of fruit and vegetables, class challenges (*other*).	
Comparator description	Usual practice or waitlist control	
Implementation outcomes	Outcome: Verbal encouragement by food staff (mean % of observations), number of fruits and vegetables on the snack cart (mean), number of fruits and vegetables students can choose (mean), fruit and vegetables rated as appealing (mean %).	
	Measure: Direct observations of the lunchroom, lunch line, food cart, and food service staff behavior.	
	Results: % of program implementation: 14% (−2% to 30%) Mean number of fruit and vegetables available: 0.64 (0.48–0.80)	
Sallis, 1997		
Study characteristics	Design: nonrandomized trial	
	Setting: Elementary schools	
	Population: California, USA	
*n* (number of participants)	Baseline: Intervention: 2 (schools); Control: 3 (schools)	Follow-up: Intervention: 2 (schools); Control: 3 (schools)
Intervention description (*implementation strategy* categorized by EPOC)	Intervention description: Sports, Play and Active Recreation for Kids (SPARK) PE was designed to be a comprehensive program for upper elementary students to increase physical activity. Designed to influence the quantity and quality of elementary PE lessons and the amount of PE through: # Lessons per week and minutes of PE per week. - Written curriculum guide identified the program philosophy and goals and included a yearly plan which was divided into instruction units with activity progressions within each unit (educational materials). - An additional 30 min per week was allocated for classroom instruction and practices in self-management activities and skills (length of consultation). - Equivalent types of equipment were provided to all seven schools, including control schools, and replacement equipment was added each year (other). - On-site support which was provided during the 3 years ensured the curriculum was followed. A PE specialist provided feedback, encouragement and direct assistance during schools visits (educational outreach visits). - Classroom teachers were trained to implement SPARK PE (educational meetings).	
Comparator description	Usual practice or waitlist control	
Implementation outcomes	Outcome: Duration (minutes) per week of physical education lessons and frequency (per week) of physical education lessons	
	Measure: Direct observation by trained assessors for one full week twice a year in each school year.	
	Results: Duration (minutes) per week of physical education lessons: 26.6 (15.3–37.9) Frequency (per week) of physical education lessons: 0.8 (0.3–1.3)	
Saunders, 2006		
Study characteristics	Design: RCT	
	Setting: High school	
	Population: South Carolina, USA	
*n* (number of participants)	Baseline: Intervention: 12 (schools); Control: 12 (schools)	Follow-up: Intervention: 12 (schools); Control: 12 (schools)
Intervention description (*implementation strategy* categorized by EPOC)	Intervention description: Focused on changing personal, social, and environmental factors related to physical activity and involved changes to the school environment and instructional programs. Instructional program components included changes in physical education and health instruction to enhance physical activity self‐efficacy and enjoyment. Schools were not required to implement a specific LEAP curriculum. Rather, to change instructional practice. The environmental strategy involved changing school practices that encouraged and supported physical activity and included changes to school health services, faculty staff health promotion, school environment, and school community linkages. - Two full-time program support staff provided (*educational outreach visits*). - Each LEAP team was headed by a LEAP champion who was usually the teacher responsible for girls PE (*local opinion leaders).* - Staff training consisted of formal workshops and one-on-one technical assistance for school personnel (*educational meetings*). - LEAP staff maintained a wide range of resources (*educational materials*). - LEAP staff worked with the LEAP champion and the LEAP team in each school to identify opportunities to enhance the environment or change school policy in support of physical activity (*local consensus approach*). - Equipment, such as hand weights, exercise bands, pedometers, for intervention schools (*other*).	
Comparator description	Usual practice or waitlist control	
Implementation outcomes	Outcome: School physical activity team, School administrator supports physical activity promotion, Emphasizes lifelong physical activity, Includes cooperative activities, School nurse counseling for physical activity, Adult modeling of physical activity through faculty/staff health promotion, Health education reinforces messages and skills taught in physical education, Community agency involvement, Family involvement.	
	Measure: The organizational assessment interview was a 22‐item interview (10–15 min) conducted by the independent process evaluator in all intervention and control schools with a school administrator, to assess organizational‐level components.	
	Results: School level policy and practice related to physical activity from the school administrators perspective	
Simons-Morton, 1988		
Study characteristics	Design: nonrandomized trial	
	Setting: Elementary schools	
	Population: Texas, USA	
*n* (number of participants)	Baseline: Intervention: 2 (schools); Control: 2 (schools)	Follow-up: Intervention: 2 (schools); Control: 2 (schools)
Intervention description (*implementation strategy* categorized by EPOC)	Intervention description: Innovations introduced into the schools included: 1) the new school lunch,) and 2) health education for healthful diet. Implementation of each of the program components required organizational changes in school programs and in the roles and practices of school personnel. - Project staff examined existing menu planning, food purchasing and recipe selection practices (*monitoring of performance*). - Practice changes in four areas purchasing, menu planning, recipes, and food preparation were identified and negotiated with the food service director and with intervention school cafeteria managers (*local consensus processes*). - Food handlers received six hours of summer in-service training conducted by the project staff in cooperation with cafeteria managers (*educational outreach visits*). - The staff dietitian served as consultant and was present in the treatment schools on a regular basis (*managerial supervision*). - Six health education modules on diet were provided, which included visual aids and teaching materials ready to be handed out to the children (*educational materials*). - Classroom modules were developed by project staff with the aid of a classroom teacher who had recently retired from the school district (*local opinion leaders*). - Children were eligible to receive token incentives, such as stickers and sweat bands, upon completion of the major learning activities (*other*).	
Comparator description	Usual practice	
Implementation outcomes	Outcome: Sodium content of school meals mg by schools, Fat content of school lunches (g).	
	Measure: Recipe analyses, based on detailed interviews with each were conducted by trained staff nutritionists and analyzed by the Nutrition Coding Center (NCC).	
	Results: Macronutrient content of school meals	
Story, 2000		
Study characteristics	Design: Cluster-RCT	
	Setting: Elementary schools	
	Population: Minnesota, USA	
*n* (number of participants)	Baseline: Intervention: 10 (schools); Control: 10 (schools)	Follow-up: Intervention: 10 (schools); Control: 10 (schools)
Intervention description (*implementation strategy* categorized by EPOC)	Intervention description: Intervention strategies were (a) point‐of‐purchase promotion of fruit and vegetable using characters and messages, (b) increasing the appeal of fruit and vegetable, (c) increasing the variety and choice of fruit and vegetable served, and (d) offering an additional fruit choice on days when baked or frozen desserts were served. - Centralized training sessions were held for food service staff from the intervention schools. It was held during a regularly scheduled school day and was conducted by the 5-a-Day Power Plus staff. Food service staff attended the teacher training for 2 hr and also attended 2 hr training after school each of the 2 intervention years (*educational meetings*). - A local producer provided some fruit and vegetable for use in classroom taste testing, home snack packs, and to expand choice in school lunch. They also provided a 30 min presentation on fruit and vegetable to each classroom (*other)*.	
Comparator description	Usual practice	
Implementation outcomes	Outcome: Mean number of fruit and vegetable choices available, Mean number of fruit and vegetable choices available, Mean % of 8 guidelines on how to offer appealing fruit and vegetable, Mean % of 8 guidelines on how to offer appealing fruit and vegetable, Mean % of 4 fruit and vegetable promotions, Mean % of 4 fruit and vegetable promotions.	
	Measure: Direct observations using trained observers and standardized protocols and instruments.	
	Results: Mean number fruit and vegetables available: 1.15 (1–1.3) % of guidelines implemented and % of promotions held: 38.4% (28.5% to 43.8%)	
Sutherland, 2017		
Study characteristics	Design: Cluster-RCT	
	Setting: Elementary schools	
	Population: NSW, Australia	
*n* (number of participants)	Baseline: Intervention: 25 (schools); Control: 21 (schools)	Follow-up: Intervention: 25 (schools); Control: 21 (schools)
Intervention description (*implementation strategy* categorized by EPOC)	The evidence‐based school physical activity program known as SCORES (Supporting Children’s Outcomes using Rewards, Exercise and Skills) was rolled out in primary schools and the implementation intervention strategies facilitated its roll out. - Schools were provided feedback on the implementation of the intervention on three occasions via email (*audit and feedback*). - Teachers were provided with resources, including lesson booklets, posters, whistles, lanyards and fundamental motor skills cards (*educational materials*). - Teachers were offered a 90 min professional learning workshop including theory and practical sessions. The workshop focused on delivery of fundamental motor skills to students, strategies to improve lesson quality through student engagement and increase students’ MVPA (*educational meeting*), - Teaching with experienced Health Promotion staff with a PE background was offered to classroom teachers in intervention schools (educational outreach visits). - A meeting with school executive was held at the commencement of intervention and a school champion nominated for each school (local opinion leader). - Additional support was provided to classroom teachers via five short video clips viewed in staff meetings, reinforcing the quality PE teaching principles (*other*).	
Comparator description:	Usual practice or waitlist control	
Implementation outcomes	Outcome: school PA policy or plan (% of schools), overall lesson quality score, recess PA (mean % of days offered), lunch PA (mean % of days offered), provision of sports equipment at recess (mean % of days offered), provision of sports equipment at lunch (mean % of days offered), provision of parent newsletters regarding PA.	
	Measure: Survey and observation	
	Results: % implementing a variety of policies and practices: 19% (16% to 22%) Physical education lesson quality score: 21.5 % of program implementation: −8% (−18% to 2%)	
Whatley Blum, 2007		
Study characteristics	Design: nonrandomized trial	
	Setting: High school	
	Population: Maine, USA	
*n* (number of participants)	Baseline: Intervention: 4 (schools); Control: 3 (schools)	Follow-up: Intervention: 4 (schools); Control: 3 (schools)
Intervention description(*implementation strategy* categorized by EPOC)	Intervention description: Implementing low‐fat, low‐sugar and portion controlled guidelines in à la carte and vending (snack and beverage) programs. - Visits by research staff to each schools food and beverage supplier to identify items that met the guidelines (*educational outreach visits*). - Suppliers who stocked vending machines were given lists of the available items and letters sent home to parents and students informing them of changes incentives. Banners were also displayed to promote healthier foods and taste testing was conducted (*educational materials*). - Modification of recipes and preparation techniques by research and food service personnel (*clinical practice guidelines*). - Food service directors were given lists of available products/vendors that met guidelines (*procurement and distribution of supplies*). - Presentations describing guidelines made to school staff (*educational meeting*). - Funding allocated to school liaison personnel (*external funding*). - A committee at each school site was created. A liaison identified at each school was responsible for establishing a committee to promote the healthy changes at the school (*local consensus approach*). - Communication between the project team and schools began in 2004 to obtain the cooperation of school administration and meet food service personnel (*other*).	
Comparator description:	Usual practice or waitlist control	
Implementation outcomes:	Outcome: % items meeting nutrient criteria in à la carte, % items meeting nutrient criteria in snack vending, % items meeting nutrient criteria in beverage vending, % items meeting nutrient and proportion criteria in à la carte, % items meeting nutrient and proportion criteria in snack vending, % items meeting nutrient and proportion criteria in beverage vending.	
	Measure: Observation and recording of items sold was taken at breakfast and lunch at cafeterias	
	Results: % of food and beverage items meeting guideline nutrient and portion criteria: 42.95% (15.7% to 60.6%)	
Wolfenden, 2017		
Study characteristics	Design: RCT	
	Setting: Elementary schools	
	Population: NSW, Australia	
*n* (number of participants)	Baseline: Intervention 35 (schools); Control: 35 (schools)	Follow-up: Intervention: 27 (schools); Control: 30 (schools)
Intervention description(*implementation strategy* categorized by EPOC)	Intervention description: Sought to increase implementation of a healthy canteen policy. Specifically, the intervention assisted schools to remove “red” and “banned” menu items from regular sale and increase the availability of “green” items to more than 50% of menu items. - Feedback menu reviews were conducted quarterly and the results were used to compile written feedback reports to the canteen manager and school principal (*audit and feedback*). - Support officers contacted canteen managers every 2 months throughout the intervention and used a continuous quality improvement framework of repeated goal setting, action planning, self-monitoring and problem-solving with canteen managers (*continuous quality improvement*). - Schools were also offered a small reimbursement to cover the costs associated with canteen manager attendance at training (*external funding*). - Printed instructional materials, sample policies/menus, planning templates and pricing guides were provided to all school canteen managers (*educational materials*). - Canteen managers, canteen staff and parent representatives were invited to attend a training workshop (5 hr) with the aim of providing education and skill development in the policy, nutrition and food label reading (*educational meeting*). - Canteen visits were conducted 1 and 3 months post-canteen manager training (*educational outreach visits*). - Meetings between support officers and canteen staff were held to discuss and reach consensus regarding the policy (*local consensus process*). - Executive support school principals were asked to communicate support for policy implementation and maintenance to teachers, parents, students and canteen managers (*local opinion leader*). - Individualized goal setting, action planning with canteen managers at different schools (*tailored interventions*). - Quarterly project newsletters communicated key messages, provided information and case studies of successful implementation approaches to common barriers (*other*).	
Comparator description	Usual practice	
Implementation outcomes	Outcome: Proportion of schools with a menu that did not contain foods or beverages restricted for sale under the policy, proportion of schools where healthy canteen items represented more than 50% of menu items.	
	Measure: Canteen menus were collected and audited by two dietitians independently.	
	Results: % implementing a variety of policies and practices: 66.6% (60.5% to 72.6%)	
Yoong, 2016		
Study characteristics	Design: RCT	
	Setting: Elementary schools	
	Population: NSW, Australia	
*n* (number of participants)	Baseline: Intervention 36 (schools); Control: 36 (schools)	Follow-up: Intervention: 29 (schools); Control: 24 (schools)
Intervention description(*implementation strategy* categorized by EPOC)	Intervention description: - Consisting of up to four menu audits together with verbal and/or written feedback delivered by Health Promotion Officers as part of routine service delivery in the study region (audit with feedback). - During feedback calls the Health Promotion Officer tailored the discussion to the needs of the Canteen Manager based on previous contact; and monitored their actions and progress toward their goals, set new goals where required, or monitored compliance (continuous quality improvement). - Schools were provided with “Fresh Tastes @ School” resources, healthy food guidelines, a menu planning template, sample policies and menus, pricing guides and a local suppliers buyer’s guide (educational materials). - The specific number of menu audits, feedback reports and calls provided was tailored depending on each school’s compliance with the guidelines and whether menu changes had occurred (tailored interventions).	
Comparator description	Usual practice	
Implementation outcomes	Outcome: Proportion of schools having a canteen menu that did not contain any “red” foods or “banned” drinks, proportion of schools having a canteen menu that contained > 50% “green” items.	
	Measure: Menu audits by trained dietitians	
	Results: % implementing a variety of policies and practices: 21.6% (15.6% to 27.5%)	
Young, 2008		
Study characteristics	Design: RCT	
	Setting: Middle schools	
	Population: Six districts across USA	
*n* (number of participants)	Baseline: Intervention: 18 (schools); Control: 18 (schools)	Follow-up: Intervention: 18 (schools); Control: 18 (schools)
Intervention description(*implementation strategy* categorized by EPOC)	Intervention description: Health education, PE, science or homeroom teachers attended workshops to teach a series of six lessons that promoted development of behavioral skills associated with physical activity. - PE teachers received instructional materials for PE lessons (*educational materials*). - PE teachers received regular on-site support to conduct lessons that encouraged active participation of girls during PE classes and to promote out-of-class physical activity (*educational outreach visits*). - Health education, PE, science or homeroom teachers attended workshops to teach a series of six lessons that promoted development of behavioral skills associated with physical activity (*educational meetings*). - Collaborations were created between schools, community agencies and staff to increase girl‐focused physical activity programs outside of PE classes (*interprofessional education*). - Program champions were directed the intervention to enhance its sustainability in the third year (*local opinion leaders*). - Intervention goals were identified for optimal intervention implementation (*local consensus processes*).	
Comparator description	Usual practice	
Implementation outcomes	Outcome: average number of physical activity programs, students encouraged for out‐of‐PE‐class physical activity (% of classes), teacher strategies to minimize management time (% classes), students provided with choices (% of classes), students encouraged for in‐class physical activity (% classes), student equipment ratio was appropriate for activity (% classes), group sizes appropriate for activity (% of classes), % of school reporting collaborations.	
	Measure: Surveys of physical activity program leaders	
	Results: % implementing a variety of policies and practices: 9.3% (−6.8% to 55.5%) Average number of physical activity programs taught: 5.1 (−0.4 to 10.6)	
Bremer, 2018		
Study characteristics	Design: nonrandomized trial	
	Setting: Middle schools	
	Population: Ontario, Canada	
*n* (number of participants)	Baseline: Intervention: 19 (classes); Control: 11 (classes)	Follow-up: Intervention: 19 (classes); Control: 11 (classes)
Intervention description(*implementation strategy* categorized by EPOC)	Intervention description: The intervention consisted of a physical activity (PA) program designed by a national organization with expertise in school-based physical activity programming and delivered in school by teachers. The program was offered to students in grades 4 through 8 and consisted of 20 min of structured PA in school for 20 consecutive weeks. - School teachers and student leaders attended a one-day workshop on how to deliver the program as part of regular school activities and were provided with instructional materials to take back to their school for program delivery (*educational meetings*). - This workshop was intended to increase teachers’ confidence to implement daily physical activity through the use of the manual and supporting resources (*educational materials*).	
Comparator description	The remaining teachers were however still expected to provide DPA to their students, as per the Ontario education curriculum.	
Implementation outcomes	Outcome: Adherence to the program, student behavior, and physical activity opportunities.	
	Measure: A 21-item questionnaire was developed for this study. Completed by the homeroom teacher at the last measurement point, it included 3 sections: adherence to the program, student behavior, and physical activity opportunities.	
	Results: Quantity physical education lessons: *t*(27) = −0.23, *p* = .82	
Cheung, 2019		
Study characteristics	Design: nonrandomized trial	
	Setting: Elementary schools	
	Population: Georgia, USA	
*n* (number of participants)	Baseline: Intervention: 71 (schools); Control: 62 (schools)	Follow-up: Intervention: 71 (schools); Control: 62 (schools)
Intervention description(*implementation strategy* categorized by EPOC)	Intervention description: Power Up for 30 (PU30) is a state-wide initiative to increase PA in school which allows tailoring of the initiative at the school level to encourage 30 min of PA outside physical education each day. - A tailored full-day training based on evidence-based strategies for increasing PA before, during (recess, in-class PA), and after school. PU30 recommended at least one administrator, one PE teacher, and one grade level chair from each school attend the training at an area school (*educational meetings*).Schools received low- and no-cost resources including exercise DVDs, PowerPoint files, and an online resource guide containing links to web-based PA videos, PA curricula, and integrated PA-academic lessons (*educational materials*).	
Comparator description	Usual practice	
Implementation outcomes	Outcome: Crude mean (standard deviation) minutes of physical activity (PA) offered per week for trained and untrained schools at baseline and follow-up.	
	Measure: School PA survey adapted from widely used school PA survey tools. PE teachers provided data regarding PE, before school and after schools PA opportunities, while grade teachers provided data regarding recess and in-class PA breaks.	
	Results: Crude mean (standard deviation) minutes of physical activity (PA) offered per week:During PE: Baseline: intervention 107.7 (4.4), control 105.6 (5.3)Follow-up: intervention 104.9 (4.3), control 105.5 (5.5)During recess: Baseline: intervention 89.8 (4.2), control 100.3 (3.9)Follow-up: intervention 98.7 (3.6), control 96.2 (3.6)In-class PA: Baseline: intervention 40.5 (2.6), control 30.4 (2.3)Follow-up: intervention 51.9 (2.5), control 36.1 (2.6)	
Egan, 2018		
Study characteristics:	Design: nonrandomized trial	
	Setting: Elementary schools	
	Population: South Eastern state, USA	
*n* (number of participants):	Baseline: Group 1: 3 (classes); Group 2: 3 (classes); Group 3: 3 (classes); Control: 3 (classes)	Follow-up: Group 1: 3 (classes); Group 2: 3 (classes); Group 3: 3 (classes); Control: 3 (classes)
Intervention description(*implementation strategy* categorized by EPOC)	Intervention description: PACES is a pilot intervention program focused on increasing children’s PA during regular school hours. It specifically targets two components: (a) physical education and (b) PA during school (i.e. opportunities to be active beyond physical education). We employed three partnership approaches (communities of practice, community-based participatory research, and service learning) based on Webster, Beets et al.’s (2015) partnership model with the aim of providing external support for the participating classroom teachers in the intervention classrooms and, subsequently, increasing the extent of MI in these classrooms. - Community-based participatory research involved a member of the research team meeting with each teacher individually to share baseline PA and MI results (*educational outreach visits*). - During the meeting, the research team identified current MI strengths and areas for improvement, collaboratively set personalized MI goals, and consider suitable resources, including those posted on the community of practice (*audit with feedback*). - The community-based participatory research strategy also included identifying each teacher’s specific MI requests and preferences (*tailored interventions*). - Schools had access to the Move for Thought website, which included educational materials, videos and links (*educational materials*).	
Comparator description	Group 1: Received the ﬁrst PACES partnership approach (community of practice); Group 2: Received the ﬁrst two approaches (community of practice and community-based participatory research); Group 3: Received all three approaches; Control: Usual practice	
Implementation outcomes	Outcome: Implementation of teacher directed transition, implementation of other movement - nonacademic, Other movement academic, Non-teacher directed transition	
	Measure: Twelve research assistants coded video records (*n* = 57) using the System for Observing Student Movement in Academic Routines and Transitions (SOSMART).	
	Results: Total implementation: Group 1: baseline 44.0, follow-up 39.13, change −4.87; Group 2: baseline 50.9, follow-up 54.27, change 3.37; Group 3: baseline 49.63, follow-up 50.73, change 1.10; Control: baseline 36.30, follow-up 35.37, change −0.93Implementation of teacher directed transition: Group 1: baseline 17.83, follow-up 14.87, change −2.97; Group 2: baseline 17.03, follow-up 20.60, change 3.57; Group 3: baseline 24.40, follow-up 21.07, change −3.33; Control: baseline 18.24, follow-up 20.20, change 1.95Implementation of other movement - nonacademic: Group 1: baseline 3.23, follow-up 2.20, change −1.00; Group 2: baseline 1.83, follow-up 4.90, change 3.07; Group 3: baseline 1.20, follow-up 12.50, change 11.33; Control: baseline, 0.59, follow-up 0.00, change -0.59Other movement academic: Group 1: baseline 2.17, follow-up 3.60, change 1.43; Group 2: baseline 0.50, follow-up 1.17, change 0.67; Group 3: baseline 1.43, follow-up 0.80, change -0.63; Control: baseline 1.18, follow-up 5.45, change 4.28Non-teacher directed transition: Group 1: baseline 20.77, follow-up 18.47, change −2.27; Group 2: baseline 31.53, follow-up 27.50, change -4.00; Group 3: baseline 22.67, follow-up 16.37, change −6.30; Control: baseline 16.16, follow-up 9.36, change −6.79	
Evenhuis, 2018		
Study characteristics	Design: nonrandomized trial	
	Setting: Elementary schools	
	Population: Netherlands	
*n* (number of participants)	Baseline: Intervention: 10 (schools); Control: 10 (schools)	Follow-up: Intervention: 10 (schools); Control: 10 (schools)
Intervention description(*implementation strategy* categorized by EPOC)	Intervention description: The intervention schools received support to implement the “Guidelines for Healthier Canteens”; i.e. an advisory meeting and report, communication materials, newsletters, an online community and a factsheet with student’s wishes/needs - Canteen advisors also measured the extent to which canteens met the Guidelines for Healthier Canteens, using the online tool “the Canteen Scan” (*audit and feedback*) - School canteen advisors provided tailored advice in an advisory meeting (*educational outreach visits*) - Schools received communication materials, newsletter with information and had access to a closed Facebook community (*educational materials*).	
Comparator description	Control schools only received the guidelines.	
Implementation outcomes	Outcome: Changes in school canteen: product availability on display, vending machines and product accessibility	
	Measure: Changes in the school canteen were assessed using the “Canteen Scan,” an online tool to measure product availability on displays and vending machines, and product accessibility	
	Results: Availability of healthier products on display: (mean) Intervention: baseline 45.80 (27.12), follow-up 77.29 (13.41)*, *p* = .007; Control: baseline 50.40 (23.00), follow-up 60.10 (15.67), *p* value not reportedAccessibility criteria: Intervention: baseline 44.00 (20.66), follow-up 60.00 (21.60), *p* = .03;Control: baseline 43.00 (20.58), follow-up 50.00 (14.91), *p* value not reported	
Farmer, 2017		
Study characteristics	Design: Cluster-RCT	
	Setting: Elementary schools	
	Population: Otago and Auckland, New Zealand	
*n* (number of participants)	Baseline: Intervention: 8 (schools); Control: 8 (schools)	Follow-up: Intervention: 8 (schools); Control: 8 (schools)
Intervention description(*implementation strategy* categorized by EPOC)	Intervention description: The researchers, playworker and school community worked together to develop a playground action plan that met the needs of each school community. Following baseline evaluations of their play space, each intervention school was provided with a list of tailored suggestions for improvements. This was speciﬁc to each school but could include the addition of more interactive play equipment, and alterations to school rules and policies that may limit risk-taking during play, with all alterations meeting playground safety standards. The research team met with each school community to ﬁnalise the plan. - Provided with funds to assist with altering school play spaces (*incentives*). - The researchers, playworker and school community worked together to develop a playground action plan that met the needs of each school community (*local consensus approach*). - School was provided with a list of tailored suggestions for improvements. This was specific to each school but could include the addition of more interactive play equipment, and alterations to school rules and policies that may limit risk-taking during play with all alterations meeting playground safety standards (*tailored interventions*).	
Comparator description	Usual practice	
Implementation outcomes	Outcome: Physical activity policies within their school (break time, using physical activity as a punishment, promotion of community activities, adequacy and availability of facilities during school/after hours, enjoyment and promotion of PA regardless of skill level, amount and quality of physical education, and safety.	
	Measure: principals completed an 18-item questionnaire assessing physical activity policies within their school. Principals indicated whether the policies were fully in place (score of 3), partially in place (2), under development (1), or not in place (0).	
	Results: School policy regarding physical activity: Follow-up: intervention 76.2% (10.4), control 76.4% (10.6), *p* = .568Provision of play opportunities: mean difference: 4.50 (95% conﬁdence interval: 1.82–7.18, *p* = .005	
Nathan, unpublished		
Study characteristics:	Design: Cluster-RCT	
	Setting: Elementary schools	
	Population: Hunter region of New South Wales, Australia	
*n* (number of participants):	Baseline: Treatment 1: 3 (schools); Treatment 2: 3 (schools); Treatment 3: 3 (schools); Control: 3 (schools)	Follow-up: Treatment 1: 3 (schools); Treatment 2: 3 (schools); Treatment 3: 3 (schools); Control: 3 (schools)
Intervention description(*implementation strategy* categorized by EPOC)	Intervention description: Three key opportunities were targeted to improve physical activity. PE teachers were supported to program PE by developing a sequential plan for each school class. Sport teachers were supported to program sufficient time for sport and maximize student activity. Teachers were supported to integrate short bouts of activity into class routines, such as energizers or active lessons. - Teachers were provided with examples of school and classroom plans that show teachers how to implement 150 min of organized activities consistent with the policy across the school week (*educational materials*). - Support officers met with all teachers once as a group in each school for 1–2 h to introduce the in-school champion and their role in implementing the intervention and as a point of support in the school; to provide instruction on the development of how to implement 150 min of organized activities consistent with the policy across the school week (*educational outreach visits*). - Support officers provided technical assistance to schools throughout the study period to support policy implementation (*centralized technical assistance*). - Support officers met with principals and school executive to communicate the importance and benefits of scheduled PA. Principals and the executive were asked to demonstrate support for implementing the policy (*mandate change*). - Each school nominated at least 2 in-school champions who were existing teachers at the school (*identify and prepare champions*). - Support officers provided in-school champions with support remotely, that is,via telephone or e-mail twice per term to support implementing the intervention (*other*).	
Comparator description	Treatment 1: PA support; Treatment 2: Lunchbox support; Treatment 3: Both PA support and lunchbox support; Control: Usual practice	
Implementation outcomes	Outcome: Mean minutes of teachers’ scheduled PA	
	Measure: at the end of each day for one school week teachers completed a paper-based log book. This included the time they engaged in all teaching activities across all subjects each day including the duration PA was provided.	
	Results: Mean minutes of teachers’ scheduled PA: Follow-up: intervention 135.95 (59.46), control 99.04 (51.83), *p* = .04	
Taylor, 2018		
Study characteristics	Design: Cluster-RCT	
	Setting: Elementary schools	
	Population: Northern California, USA	
*n* (number of participants)	Baseline: Intervention: 1 (schools); Control: 1 (schools)	Follow-up: Intervention: 1 (schools); Control: 1 (schools)
Intervention description(*implementation strategy* categorized by EPOC)	Intervention description: Incorporates 5 program objectives: (1) increase nutrition knowledge and use of science processing skills among fourth-grade children; (2) promote availability, consumption, and enjoyment of fruits and vegetables in the school environment; (3) improve dietary patterns and encourage physical activity; (4) foster positive changes in the school environment; and (5) facilitate development of an infrastructure to sustain the program. - The school district was provided $3000 to increase procurement of regionally grown produce for use in the National School Lunch Program (*incentives*). - Program activities included 20 hr of classroom education using an inquiry-based, garden-enhanced nutrition curriculum (*educational outreach visits*). - Take-home activities, materials and family newsletters (*educational materials*).	
Comparator description	Usual practice or waitlist control	
Implementation outcomes	Outcome: Fruit and vegetable availability	
	Measure: Based on produce expenditures and variety for use in the schools. Procurement records were used to determine how many different types of fruits and vegetables were offered	
	Results: Fruit offered daily by schools: Baseline: 4.33 ± 0.82 control, 4.80 ± 1.10 intervention, *p* = .44; Follow-up: 4.17 ± 0.75 control, 4.17 ± 0.98, *p* = 1.00Vegetables offered daily by schools: Baseline:2.67 ± 0.52 control, 5.40 ± 1.95 intervention, *p* = .03; Follow-up: 3.00 ± 0.89 control, 8.33 ± 0.82 intervention, *p* < .001	
Waters, 2017		
Study characteristics	Design: Cluster-RCT	
	Setting: Elementary schools	
	Population: Northern California, USA	
*n* (number of participants)	Baseline: Intervention: 12 (schools); Control: 12 (schools)	Follow-up: Intervention: 12 (schools); Control: 10 (schools)
Intervention description(*implementation strategy* categorized by EPOC)	Schools were supported to develop fun “n healthy programs according to the fixed requirement of a whole school combined focus on increasing fruit, vegetable and water consumption, increasing physical activity and encouraging self-esteem in children. - The school community determined the exact content of the program strategies (*tailored interventions and local consensus approach*) - Community Development Workers acted as knowledge brokers, providing information and guiding schools’ customized development of intervention program strategies and their efforts to resource them (*educational outreach visits and educational materials*).	
Comparator description	Usual practice	
Implementation outcomes	Outcome: Proportion of schools with written physical activity, healthy eating and canteen policies	
	Measure: School principals were asked to report on whether their school had policies relating to physical activity and canteen	
	Results: Proportion of schools with physical activity policy: Control: baseline 7 (70%), follow-up 6 (60%); Intervention: baseline 8 (66.6%), follow-up 11 (91.7%)Proportion of schools with healthy eating policy: Control: follow-up 2 (20%); Intervention: follow-up 9 (75%)Proportion of schools with canteen policy: Control: baseline 4 (40%), follow-up 6 (60%); Intervention: baseline 2 (16.7%), follow-up 3 (25%)	

## References

[1] Global Disease Collaborators. Health effects of dietary risks in 195 countries, 19902017: A systematic analysis for the Global Burden of Disease Study 2017. 2019;393(10184):1958–1972. 10.1016/S0140-6736(19)30041-8 PMC6899507 30954305

[2] Burrows TL , Whatnall MC, Patterson AJ, Hutchesson MJ. Associations between dietary intake and academic achievement in college students: A systematic review. Healthcare (Basel). 2017;5(4):60. 10.3390/healthcare5040060 PMC5746694 28946663

[3] O’Neil A , Quirk SE, Housden S, et al. Relationship between diet and mental health in children and adolescents: A systematic review. Am J Public Health. 2014;104(10):e31–e42. 10.2105/AJPH.2014.302110 PMC4167107 25208008

[4] Lubans D , Richards J, Hillman C, et al. Physical activity for cognitive and mental health in youth: A systematic review of mechanisms. Pediatrics. 2016;138(3):e20161642. 27542849 10.1542/peds.2016-1642

[5] Singh A , Uijtdewilligen L, Twisk JW, van Mechelen W, Chinapaw MJ. Physical activity and performance at school: A systematic review of the literature including a methodological quality assessment. Arch Pediatr Adolesc Med. 2012;166(1):49–55. 22213750 10.1001/archpediatrics.2011.716

[6] Donnelly JE , Hillman CH, Castelli D, et al. Physical Activity, fitness, cognitive function, and academic achievement in children: A systematic review. Med Sci Sports Exerc. 2016;48(6):1197–1222. 27182986 10.1249/MSS.0000000000000901 PMC4874515

[7] OECD. The Heavy Burden of Obesity: The Economics of Prevention. Paris, France: OECD Publishing; 2019.

[8] Swinburn BA , Sacks G, Hall KD, et al. The global obesity pandemic: Shaped by global drivers and local environments. Lancet. 2011;378(9793):804–814. 21872749 10.1016/S0140-6736(11)60813-1

[9] World Health Organization. Population based approaches to childhood obesity prevention. 2012. Available at www.who.int/dietphysicalactivity/childhood/WHO_new_childhoodobesity_PREVENTION_27nov_HR_PRINT_OK.pdf. Accessibility verified May 12, 2020.

[10] World Health Organization. Population-based approaches to childhood obesity prevention. 2012. Available at https://www.who.int/dietphysicalactivity/childhood/approaches/en/. Accessibility verified May 12, 2020.

[11] World Health Organization. Report of the Commission on Ending Childhood Obesity. Implementation Plan: Executive Summary. Geneva, Switzerland: World Health Organization; 2017.

[12] Demetriou Y , Höner O. Physical activity interventions in the school setting: A systematic review. Psychol Sport Exerc. 2012;13(2):186–196.

[13] Brown T , Summerbell C. Systematic review of school-based interventions that focus on changing dietary intake and physical activity levels to prevent childhood obesity: An update to the obesity guidance produced by the National Institute for Health and Clinical Excellence. Obes Rev. 2009;10(1):110–141. 18673306 10.1111/j.1467-789X.2008.00515.x

[14] Liu Z , Xu HM, Wen LM, et al. A systematic review and meta-analysis of the overall effects of school-based obesity prevention interventions and effect differences by intervention components. Int J Behav Nutr Phys Act. 2019;16(1):95. 31665040 10.1186/s12966-019-0848-8 PMC6819386

[15] Australian Government Department of Health and Ageing. National Healthy School Canteens Pocket Guide. 2013. Available at https://www1.health.gov.au/internet/main/publishing.nsf/Content/nhsc-pocket-guide. Accessibility verified May 12, 2020.

[16] Public Health England. What works in schools and colleges to increase physical activity? 2020. Available at https://assets.publishing.service.gov.uk/. Accessibility verified May 12, 2020.

[17] Society of Health and Physical Educators. National Guidelines 2019. Available at https://www.shapeamerica.org/. Accessibility verified May 12, 2020.

[18] Government of Canada. Healthy eating at school 2019. Available at https://food-guide.canada.ca/en/tips-for-healthy-eating/school/. Accessibility verified May 12, 2020.

[19] NSW Government. Sport and Physical Activity Policy 2020. Available at https://policies.education.nsw.gov.au/policy-library/policies/sport-and-physical-activity-policy. Accessibility verified May 14, 2020.

[20] Harrington DM , Belton S, Coppinger T, et al. Results from Ireland’s 2014 Report Card on physical activity in children and youth. J Phys Act Health. 2014;11(suppl 1):S63–S68. 25426916 10.1123/jpah.2014-0166

[21] Yoong SL , Nathan NK, Wyse RJ, et al. Assessment of the school nutrition environment: A study in australian primary school canteens. Am J Prev Med. 2015;49(2):215–222. 26091931 10.1016/j.amepre.2015.02.002

[22] Chriqui JF , Resnick EA, Schneider L, Schermbeck R. School District Wellness Policies: Evaluating Progress and Potential for Improving Children’s Health Five Years After the Federal Mandate. Chicago, IL: Bridging the Gap Program, Health Policy Center, Institute for Health Research and Policy, University of Illinois at Chicago; 2013.

[23] Glasgow RE , Vinson C, Chambers D, Khoury MJ, Kaplan RM, Hunter C. National Institutes of Health approaches to dissemination and implementation science: Current and future directions. Am J Public Health. 2012;102(7):1274–1281. 22594758 10.2105/AJPH.2012.300755 PMC3478005

[24] Brownson RC , Colditz GA, Proctor EK. Dissemination and Implementation Research in Health: Translating Science to Practice. London: Oxford University Press; 2012.

[25] Wolfenden L , Nathan NK, Sutherland R, et al. Strategies for enhancing the implementation of school‐based policies or practices targeting risk factors for chronic disease. Cochrane Database Syst Rev. 2017;11(11):CD011677. 29185627 10.1002/14651858.CD011677.pub2 PMC6486103

[26] Bremer E , Graham JD, Veldhuizen S, Cairney J. A program evaluation of an in-school daily physical activity initiative for children and youth. BMC Public Health. 2018;18(1):1023. 30115054 10.1186/s12889-018-5943-2 PMC6097410

[27] Waters E , Gibbs L, Tadic M, et al. Cluster randomised trial of a school-community child health promotion and obesity prevention intervention: Findings from the evaluation of fun ‘n healthy in Moreland! BMC Public Health. 2017;18(1):92. 28774278 10.1186/s12889-017-4625-9 PMC5543738

[28] Egan CA . Two studies of partnership approaches to comprehensive school physical activity programming: A process evaluation and a case study. Diss Abstr Int Section A Humanit Soc Sci. 2018;78(11-A(E)):64–112.

[29] Liberati A , Altman DG, Tetzlaff J, et al. The PRISMA statement for reporting systematic reviews and meta-analyses of studies that evaluate healthcare interventions: Explanation and elaboration. BMJ. 2009;339:b2700. 19622552 10.1136/bmj.b2700 PMC2714672

[30] Schillinger D. An Introduction to Effectiveness, Dissemination and Implementation Research. UCSF Clinical and Translational Science Institute (CTSI) Resource Manuals and Guides to Community-Engaged Research. San Francisco, CA: University of California; 2010.

[31] Australian Institute of Health Welfare. Australia’s Children. Canberra, Australia: AIHW; 2020.

[32] Effective Practice and Organisation of Care. The EPOC Taxonomy of Health Systems Interventions. EPOC Resources for Review Authors. Oslo, Finland Norwegian Knowledge Centre for the Health Services. 2016. Available at epoc.cochrane.org/epoc-taxonomy. Accessibility verified May 26, 2020.

[33] Higgins JPT , Green S, eds. Cochrane Handbook for Systematic Reviews of Interventions Version 5.1.0. Chichester: The Cochrane Collaboration; 2011.

[34] Bilandzic A , Fitzpatrick T, Rosella L, Henry D. Risk of bias in systematic reviews of non-randomized studies of adverse cardiovascular effects of thiazolidinediones and cyclooxygenase-2 inhibitors: Application of a new cochrane risk of bias tool. PLoS Med. 2016;13(4):e1001987. 27046153 10.1371/journal.pmed.1001987 PMC4821619

[35] Wolfenden L , Barnes C, Jones J, et al. Strategies to improve the implementation of healthy eating, physical activity and obesity prevention policies, practices or programmes within childcare services. Cochrane Database Syst Rev. 2020;2(2):CD011779. 32036618 10.1002/14651858.CD011779.pub3 PMC7008062

[36] Wolfenden L , Goldman S, Stacey FG, et al. Strategies to improve the implementation of workplace‐based policies or practices targeting tobacco, alcohol, diet, physical activity and obesity. Cochrane Database Syst Rev. 2018;11(11):CD012439. 30480770 10.1002/14651858.CD012439.pub2 PMC6362433

[37] Nathan NK , Sutherland RL, Hope K, et al. Implementation of a school physical activity policy improves student physical activity levels: Outcomes of a cluster-randomized controlled trial. J Phys Act Health. 2020;17(10):1009–1018. 32919383 10.1123/jpah.2019-0595

[38] Cheung P , Franks P, Kramer M, et al. Impact of a Georgia elementary school-based intervention on physical activity opportunities: A quasi-experimental study. J Sci Med Sport. 2019;22(2):191–195. 30126698 10.1016/j.jsams.2018.07.015 PMC6637426

[39] Egan CA , Webster C, Weaver RG, et al. Partnerships for Active Children in Elementary Schools (PACES): First year process evaluation. Eval Program Plann. 2018;67:61–69. 29227866 10.1016/j.evalprogplan.2017.12.002

[40] Evenhuis IJ , Vyth EL, Jacobs S, Veldhuis L, Seidell JC, Renders CM. Does implementation of guidelines for healthier school canteens result in changes in canteens and healthier purchase behaviour of students? Obesity Facts. 2018;11(suppl 1):119–120.

[41] Farmer VL , Williams SM, Mann JI, Schofield G, McPhee JC, Taylor RW. The effect of increasing risk and challenge in the school playground on physical activity and weight in children: A cluster randomised controlled trial (PLAY). Int J Obes (Lond). 2017;41(5):793–800. 28186099 10.1038/ijo.2017.41

[42] Taylor JC , Zidenberg-Cherr S, Linnell JD, Feenstra G, Scherr RE. Impact of a multicomponent, school-based nutrition intervention on students’ lunchtime fruit and vegetable availability and intake: A pilot study evaluating the Shaping Healthy Choices Program. J Hunger Environ Nutr. 2018;13(3):415–428.

[43] Alaimo K , Carlson JJ, Pfeiffer KA, et al. Project FIT: A school, community and social marketing intervention improves healthy eating among low-income elementary school children. J Community Health. 2015;40(4):815–826. 25940937 10.1007/s10900-015-0005-5

[44] Cunningham-Sabo L , Lohse B, Smith S, et al. Fuel for Fun: A cluster-randomized controlled study of cooking skills, eating behaviors, and physical activity of 4th graders and their families. BMC Public Health. 2016;16:444. 27230565 10.1186/s12889-016-3118-6 PMC4882848

[45] Delk J , Harrell MB, Fakhouri THI, Muir KA, Perry CL. Implementation of a computerized tablet-survey in an adolescent large-scale, school-based study. J Sch Health. 2017;87(7):506–512. 28580672 10.1111/josh.12521 PMC5485855

[46] Delk J , Springer AE, Kelder SH, Grayless M. Promoting teacher adoption of physical activity breaks in the classroom: findings of the Central Texas CATCH Middle School Project. J Sch Health. 2014;84(11):722–730. 25274172 10.1111/josh.12203

[47] French SA , Story M, Fulkerson JA, Hannan P. An environmental intervention to promote lower-fat food choices in secondary schools: Outcomes of the TACOS Study. Am J Public Health. 2004;94(9):1507–1512. 15333303 10.2105/ajph.94.9.1507 PMC1448482

[48] Heath EM , Coleman KJ. Evaluation of the institutionalization of the coordinated approach to child health (CATCH) in a U.S./Mexico border community. Health Educ Behav. 2002;29(4):444–460. 12137238 10.1177/109019810202900405

[49] Hoelscher DM , Springer AE, Ranjit N, et al. Reductions in child obesity among disadvantaged school children with community involvement: The Travis County CATCH Trial. Obesity (Silver Spring). 2010;18(suppl 1):S36–S44. 20107459 10.1038/oby.2009.430

[50] Lytle LA , Kubik MY, Perry C, Story M, Birnbaum AS, Murray DM. Influencing healthful food choices in school and home environments: Results from the TEENS study. Prev Med. 2006;43(1):8–13. 16697452 10.1016/j.ypmed.2006.03.020

[51] Mobley CC , Stadler DD, Staten MA, et al.; HEALTHY Study Group. Effect of nutrition changes on foods selected by students in a middle school-based diabetes prevention intervention program: The HEALTHY experience. J Sch Health. 2012;82(2):82–90. 22239133 10.1111/j.1746-1561.2011.00670.x PMC3261591

[52] Nathan N , Wolfenden L, Bell AC, et al. Effectiveness of a multi-strategy intervention in increasing the implementation of vegetable and fruit breaks by Australian primary schools: A non-randomized controlled trial. BMC Public Health. 2012;12:651. 22889085 10.1186/1471-2458-12-651 PMC3490882

[53] Nathan N , Yoong SL, Sutherland R, et al. Effectiveness of a multicomponent intervention to enhance implementation of a healthy canteen policy in Australian primary schools: A randomised controlled trial. Int J Behav Nutr Phys Act. 2016;13(1):106. 27717393 10.1186/s12966-016-0431-5 PMC5054617

[54] Naylor PJ , Macdonald HM, Reed KE, McKay HA. Action Schools! BC: A socioecological approach to modifying chronic disease risk factors in elementary school children. Prev Chronic Dis. 2006;3(2):A60. 16539801 PMC1563946

[55] Perry CL , Bishop DB, Taylor GL, et al. A randomized school trial of environmental strategies to encourage fruit and vegetable consumption among children. Health Educ Behav. 2004;31(1):65–76. 14768658 10.1177/1090198103255530

[56] Perry CL , Sellers DE, Johnson C, et al. The Child and Adolescent Trial for Cardiovascular Health (CATCH): Intervention, implementation, and feasibility for elementary schools in the United States. Health Educ Behav. 1997;24(6):716–735. 9408786 10.1177/109019819702400607

[57] Sallis JF , McKenzie TL, Alcaraz JE, Kolody B, Faucette N, Hovell MF. The effects of a 2-year physical education program (SPARK) on physical activity and fitness in elementary school students. Sports, Play and Active Recreation for Kids. Am J Public Health. 1997;87(8):1328–1334. 9279269 10.2105/ajph.87.8.1328 PMC1381094

[58] Story M , Mays RW, Bishop DB, et al. 5-a-day Power Plus: process evaluation of a multicomponent elementary school program to increase fruit and vegetable consumption. Health Educ Behav. 2000;27(2):187–200. 10768800 10.1177/109019810002700205

[59] Simons-Morton BG , Parcel GS, O’Hara NM. Implementing organizational changes to promote healthful diet and physical activity at school. Health Educ Q. 1988;15(1):115–130. 3366583 10.1177/109019818801500110

[60] Sutherland RL , Nathan NK, Lubans DR, et al. An RCT to facilitate implementation of school practices known to increase physical activity. Am J Prev Med. 2017;53(6):818–828. 29051015 10.1016/j.amepre.2017.08.009

[61] Whatley Blum JE , Davee AM, Devore RL, et al. Implementation of low-fat, low-sugar, and portion-controlled nutrition guidelines in competitive food venues of Maine public high schools. J Sch Health. 2007;77(10):687–693. 18076414 10.1111/j.1746-1561.2007.00252.x

[62] Wolfenden L , Nathan N, Janssen LM, et al. Multi-strategic intervention to enhance implementation of healthy canteen policy: A randomised controlled trial. Implement Sci. 2017;12(1):6. 28077151 10.1186/s13012-016-0537-9 PMC5225642

[63] Yoong SL , Nathan N, Wolfenden L, et al. CAFÉ: a multicomponent audit and feedback intervention to improve implementation of healthy food policy in primary school canteens: A randomised controlled trial. Int J Behav Nutr Phys Act. 2016;13(1):126. 27919261 10.1186/s12966-016-0453-z PMC5139098

[64] Young DR , Steckler A, Cohen S, et al. Process evaluation results from a school- and community-linked intervention: The Trial of Activity for Adolescent Girls (TAAG). Health Educ Res. 2008;23(6):976–986. 18559401 10.1093/her/cyn029 PMC2583909

[65] Taylor SL , Noonan RJ, Knowles ZR, et al. Evaluation of a pilot school-based physical activity clustered randomised controlled trial-active schools: skelmersdale. Int J Environ Res Public Health. 2018;15(5):17. 10.3390/ijerph15051011 PMC5982050 29772839

[66] Saunders RP , Ward D, Felton GM, Dowda M, Pate RR. Examining the link between program implementation and behavior outcomes in the lifestyle education for activity program (LEAP). Eval Program Plann. 2006;29(4):352–364. 17950863 10.1016/j.evalprogplan.2006.08.006

[67] Evenhuis IJ , Jacobs SM, Vyth EL, et al. The effect of supportive implementation of healthier canteen guidelines on changes in Dutch school canteens and student purchase behaviour. Nutrients. 2020;12(8):2419. 10.3390/nu12082419 PMC7468849 32806649

[68] Bell LL. *Grandparents Raising Grandchildren in the 21st Century* [PhD dissertation]. Ann Arbor, MI: Mississippi State University; 2018.

[69] Love R , Adams J, van Sluijs EMF. Are school-based physical activity interventions effective and equitable? A systematic review and meta-analysis of cluster randomised controlled trials. *Lancet*. 2018;392:S53. 10.1111/obr.12823 PMC6563481 30628172

[70] World Health Organisation. School and youth health 2020. Available at https://www.who.int/school_youth_health/gshi/hps/en/. Accessibility verified May 26, 2020.

[71] National Health and Medical Research Council. Nutrient Reference Values Canberra2020. Available at https://www.nrv.gov.au/. Accessibility verified May 14, 2020.

[72] Hill JO , Wyatt HR, Reed GW, Peters JC. Obesity and the environment: Where do we go from here? Science. 2003;299(5608):853–855. 12574618 10.1126/science.1079857

[73] Braun HA , Kay CM, Cheung P, Weiss PS, Gazmararian JA. Impact of an elementary school-based intervention on physical activity time and aerobic capacity, Georgia, 2013–2014. Public Health Rep. 2017;132(2_suppl):24S–32S. 29136482 10.1177/0033354917719701 PMC5692171

[74] Gaglio B , Shoup JA, Glasgow RE. The RE-AIM framework: A systematic review of use over time. Am J Public Health. 2013;103(6):e38–e46. 10.2105/AJPH.2013.301299 PMC3698732 23597377

[75] Glasgow RE , Huebschmann AG, Brownson RC. Expanding the CONSORT Figure: Increasing transparency in reporting on external validity. Am J Prev Med. 2018;55(3):422–430. 30033029 10.1016/j.amepre.2018.04.044

[76] Powell BJ , Waltz TJ, Chinman MJ, et al. A refined compilation of implementation strategies: Results from the Expert Recommendations for Implementing Change (ERIC) project. Implement Sci. 2015;10(1):21. 25889199 10.1186/s13012-015-0209-1 PMC4328074

